# OptoLacI: optogenetically engineered lactose operon repressor LacI responsive to light instead of IPTG

**DOI:** 10.1093/nar/gkae479

**Published:** 2024-06-11

**Authors:** Meizi Liu, Zuhui Li, Jianfeng Huang, Junjun Yan, Guoping Zhao, Yanfei Zhang

**Affiliations:** Key Laboratory of Engineering Biology for Low-Carbon Manufacturing, Tianjin Institute of Industrial Biotechnology, Chinese Academy of Sciences, Tianjin 300308, China; National Center of Technology Innovation for Synthetic Biology, Tianjin 300308, China; Haihe Laboratory of Synthetic Biology, Tianjin 300308, China; Key Laboratory of Engineering Biology for Low-Carbon Manufacturing, Tianjin Institute of Industrial Biotechnology, Chinese Academy of Sciences, Tianjin 300308, China; National Center of Technology Innovation for Synthetic Biology, Tianjin 300308, China; School of Biological Engineering, Tianjin University of Science & Technology, Tianjin 300457, China; Key Laboratory of Engineering Biology for Low-Carbon Manufacturing, Tianjin Institute of Industrial Biotechnology, Chinese Academy of Sciences, Tianjin 300308, China; National Center of Technology Innovation for Synthetic Biology, Tianjin 300308, China; Key Laboratory of Engineering Biology for Low-Carbon Manufacturing, Tianjin Institute of Industrial Biotechnology, Chinese Academy of Sciences, Tianjin 300308, China; National Center of Technology Innovation for Synthetic Biology, Tianjin 300308, China; College of Life Sciences, University of Chinese Academy of Sciences, Beijing 101408, China; National Center of Technology Innovation for Synthetic Biology, Tianjin 300308, China; CAS-Key Laboratory of Synthetic Biology, CAS Center for Excellence in Molecular Plant Sciences, Institute of Plant Physiology and Ecology, Chinese Academy of Sciences, Shanghai 200032, China; Key Laboratory of Engineering Biology for Low-Carbon Manufacturing, Tianjin Institute of Industrial Biotechnology, Chinese Academy of Sciences, Tianjin 300308, China; National Center of Technology Innovation for Synthetic Biology, Tianjin 300308, China

## Abstract

Optogenetics’ advancement has made light induction attractive for controlling biological processes due to its advantages of fine-tunability, reversibility, and low toxicity. The lactose operon induction system, commonly used in *Escherichia coli*, relies on the binding of lactose or isopropyl β-d-1-thiogalactopyranoside (IPTG) to the lactose repressor protein LacI, playing a pivotal role in controlling the lactose operon. Here, we harnessed the light-responsive light-oxygen-voltage 2 (LOV2) domain from *Avena sativa* phototropin 1 as a tool for light control and engineered LacI into two light-responsive variants, OptoLacI^L^ and OptoLacI^D^. These variants exhibit direct responsiveness to light and darkness, respectively, eliminating the need for IPTG. Building upon OptoLacI, we constructed two light-controlled *E. coli* gene expression systems, Opto*E.coli*^Light^ system and Opto*E.coli*^Dark^ system. These systems enable bifunctional gene expression regulation in *E. coli* through light manipulation and show superior controllability compared to IPTG-induced systems. We applied the Opto*E.coli*^Dark^ system to protein production and metabolic flux control. Protein production levels are comparable to those induced by IPTG. Notably, the titers of dark-induced production of 1,3-propanediol (1,3-PDO) and ergothioneine exceeded 110% and 60% of those induced by IPTG, respectively. The development of OptoLacI will contribute to the advancement of the field of optogenetic protein engineering, holding substantial potential applications across various fields.

## Graphical Abstract

**Graphical Abstract:**
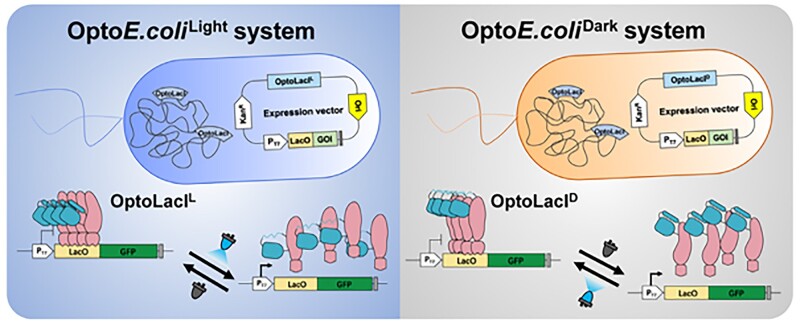

## Introduction

*Escherichia coli* is a highly favored host for numerous biotechnology applications, owing to its well-characterized genetic background, ease of manipulation, and rapid expression cycle (1–3). Over the past few decades, the lactose operon-based inducible system has gained significant prominence among the various inducible systems utilized in *E. coli*. In the absence of the chemical inducer isopropyl-β-d-1-thiogalactopyranoside (IPTG), the repressor protein LacI binds to the operator sequence *lacO*, effectively blocking the RNA polymerase from transcribing the target genes. Conversely, when IPTG and LacI combine, LacI dissociates from the *lacO*, initiating transcription of the target genes (4,5). However, drawbacks such as high cost of IPTG, potential toxicity, and irreversibility limit its application in the production of low and medium-value compounds (6). Consequently, researchers have endeavored to explore alternative methods that can overcome these limitations.

Light-inducible systems offer several advantages over conventional chemical induction methods, including low toxicity, low cost, excellent reversibility, and high spatial and temporal resolution. These characteristics make them ideal tools for gene induction, cell function research, and disease therapy (7–11). Various optogenetic systems have been developed in *E. coli*, including some based on bacterial two-component regulatory systems (TCSs), which require more than two components and additional chromophores or corresponding chromophore synthesis genes (12,13), such as the OptoLAC system, which is built upon the pDawn system, employs a series of circuits to optogenetically regulate the expression of the lactose repressor protein LacI. This enables the engineering of a metabolic pathway that utilizes blue light, rather than IPTG, to control chemical production in laboratory-scale bioreactors (14). Due to the complexity of TCSs, some signal-component regulatory systems have been developed based on a single transcription factor (15,16), such as the BLADE and eLightOn systems (7,10). The BLADE system achieves blue light inducible dimerization of the transcriptional regulator AraC in *E. coli* by replacing its dimerization domain with the light-induced Vivid (VVD) domain (10). The eLightOn system is based on the synthetic light-switchable repressor LexRO, under blue light irradiation, the LexRO dimer dissociates, leading to dissociation from the operator sequence and initiation of gene expression. The system is characterized by a high on/off ratio, high activation levels, and fast activation kinetics (7).

With the advancement of optogenetics, an increasing number of photosensitive elements have been developed and applied in cell engineering, finding utility in various biological chassis (4,8,12,17,18). Among the diverse photosensory modules, the light-oxygen-voltage-sensing domain 2 (AsLOV2) of *Avena sativa* phototropin 1 emerges as one of the most popular photosensitive elements. The distinctive features of the LOV2 domain include its flavin mononucleotide (FMN) cofactor, an endogenous chromophore present in almost all species, and its compact size (∼12 kDa) (19,20). The relatively close distance between its termini in the dark state facilitates the direct insertion of the LOV2 domain into the exposed loops of the target protein, making it highly versatile for engineering and various applications (21). Employing the AsLOV2 domain as a light-controllable tool, designing and modifying LacI to respond directly to light instead of IPTG, and developing a genetically inducible expression system that can be directly controlled by light holds promise for achieving light control over cell metabolism, protein production, and chemical product synthesis, and more. This light-controlled technology is not only applicable to industrial production but is also important for fundamental research (8,22,23).

In this study, we strategically inserted the LOV2 domain into LacI, generating OptoLacI variants capable of direct control by blue light. Leveraging OptoLacI, we developed two light-controlled gene expression systems in *E. coli*: Opto*E.coli*^Light^ system and Opto*E.coli*^Dark^ system. These systems facilitate bifunctional light-controlled gene expression in *E. coli* with excellent light control performance. Moreover, they hold immense potential for applications in synthetic biology and metabolic engineering, promising to further advance the utilization of optogenetics in these fields.

## Materials and methods

### General molecular biology techniques

The oligonucleotides used in this study were obtained from GENEWIZ (Azenta Life Sciences, Tianjin, China) (Supplementary Table S1). Genes from *E. coli* were amplified from genomic DNA of *E. coli* MG1655 by polymerase chain reaction (PCR). Genes from other organisms, including genes encoding AsLOV2(408–543), cpLOV27, and sfGFP, were codon optimized for *E. coli* and synthesized by GENEWIZ (Azenta Life Sciences, Tianjin, China) (Supplementary Table S2). DNA construction was performed using standard restriction-enzyme digestion and ligation cloning and isothermal assembly (Gibson Assembly) (24). *Escherichia coli* DH5α was used for routine transformations and plasmid production. All of the constructed plasmids (Supplementary Table S3) were verified by DNA sequencing (GENEWIZ from Azenta Life Sciences, Tianjin, China).

### Plasmids construction

#### Construction of plasmids for OptoLacI screening and optimization

We cloned the super-fold green fluorescent protein (sfGFP) gene into the commercially available pET-28a vector using the NheI and XhoI restriction sites to construct the plasmid pZH37 (Control-1). The LacI and *LacO* remained unaltered during this process, maintaining their original sequences within the pET-28a vector. The screening plasmid, pZH35 (Control-2), designed for screening OptoLacI variants, was constructed by integrating the gene encoding super-fold green fluorescent protein (sfGFP) into plasmid pET-28a using the NheI and XhoI restriction sites. Additionally, the original tryptophan (W) at position 220 of LacI was mutated to phenylalanine (F), and the original 1× *lacO1* sequence on the plasmid was replaced with a 3x *lacO1* sequence. Subsequently, the encoded sequence of AsLOV2(408–543) or cpLOV27 was inserted into different candidate sites of LacI^W220F^ on pZH35 through Gibson assembly, constructing a series of plasmids for screening. Site saturation mutagenesis was used to substitute LacI^K84^. Primer-induced mutagenesis was employed to change the copy numbers of *lacO1*.

#### Construction of plasmids for protein production

The pZH99 derived plasmid pML308 (carrying OptoLacI^D2^ and 4× *lacO1*) served as the basis to construct the protein production plasmids. The sfGFP gene on pML308 was replaced with gene of interest through standard restriction-enzyme digestion and ligation cloning using the NheI and XhoI restriction sites. Additionally, to construct the plasmids for use in the IPTG induced system, we cloned the genes encoding the target proteins into the commercially available pET-28a vector using the NheI and XhoI restriction sites. The resulting plasmids were utilized for overexpressing specific proteins, such as alkaline protease from *Bacillus licheniformis* SHG10 (pML333), PETase from *Ideonella sakaiensis* (pML351), and glucose dehydrogenase (GDH) from *E. coli* (pML353).

#### Construction of plasmids for controlling metabolic fluxes

Plasmid pJH10, designed for 1,3-propanediol production, was constructed by cloning the genes of glycerol dehydrogenase (*KpdhaB*), glycerol dehydrogenase reactivase (*KpgdrAB*) from the *Klebsiella pneumoniae* genome, alcohol dehydrogenase (*EcyqhD*) from the genome of *E. coli* MG1655 into the expression vector pCDFDuet through Gibson assembly. Plasmid pJH11 for 1,3-propanediol production was constructed by substituting the gene of LacI on pJH10 with OptoLacI^D^ through Gibson assembly. Similarly, plasmids pJY165 and pJY111, designed for ergothioneine (EGT) production, were constructed by cloning the synthesized genes of S-adenosylmethionine-dependent methyltransferase (EgtD) and C-S lyase (EgtE) from *Mycobacterium smegmatis*, and methyltransferase-sulfoxide synthase (Egt1) from *Neurospora crassa* into the dark-induced expression vector pML291 (carrying OptoLacI^D2^ and 1× *lacO1*) and the pET-28a vector, respectively, using Gibson assembly.

### Genome editing of *E. coli*

CRISPR-Cas9-based genome editing tool pEcCas/pTargetF system (25), was employed to replace the two copies of the *LacI* gene (*LacI1* and *LacI2*) present in the chromosomes of *E. coli* BL21 (DE3) with OptoLacI^D^, OptoLacI^D2^, and OptoLacI^L^, respectively. We firstly constructed the pTargetF-*lacI* plasmids (pZH83 and pML340) targeting to the *lacI* while avoiding interference with OptoLacI^D^, OptoLacI^D2^, and OptoLacI^L^. Subsequently, we constructed the patch plasmids, each containing the gene of OptoLacI^L^, OptoLacI^D^, and OptoLacI^D2^, respectively, flanked by the 500-bp upstream and 500-bp downstream homology arms of *LacI1* and *LacI2*, respectively, on the genome (pZH76, pZH78, pML318, pML320, pML347, and pML348, Supplementary Table S3).

The linearized patch fragments from plasmids pZH76 and pZH78, along with the pTargetF-*lacI* plasmid pZH83, were co-transformed into BL21(DE3) electrocompetent cells carrying the plasmid pEcCas to construct the OptoBL21^Dark^ strain. Similarly, the linearized patch fragments from plasmids pML318 and pML320 (both carrying OptoLacI^D2^), along with the pTargetF plasmid pML340, were co-transformed into BL21(DE3)-Amp-Cm electrocompetent cells carrying the plasmid pEcCas, to construct the OptoBL21^Dark-2^ strain. BL21(DE3)-Amp-Cm strain was pre-constructed by substitution of the two copies of the wild-type *lacI* in the BL21(DE3) genome with the ampicillin resistance gene (*bla*) and the chloramphenicol resistance gene (*cat*), respectively. The linearized patch fragments of plasmids pML347 and pML348 (both carrying OptoLacI^L^), along with the pTargetF plasmid pML340, were co-transformed into BL21(DE3)-Amp-Cm electrocompetent cells carrying the plasmid pEcCas to construct the OptoBL21^Light^ strain. For detailed information on mutant strain screening and plasmid curing methods, please refer to previous reports (25,26).

### Growth and measurement of bacterial cultures

Freshly transformed single colonies were selected and inoculated into 10 ml LB medium containing 50 μg/ml kanamycin. The incubated cultures were incubated at 37 °C in an orbital shaker (Zhichu Instrument Co., Ltd, Shanghai, China) under blue light condition (60 μmol m^−2^s^−1^) or dark condition for a duration of 8–10 h, depending on whether they belonged to the OptoBL21^Dark^ strains or OptoBL21^Light^ strains, respectively. Subsequently, the cultures were inoculated into 1 ml LB medium supplemented with kanamycin (50 μg/ml), at a 1% inoculum, in two 24-well plates (NEST, 702001, China). One plate was incubated at 37 °C under dark condition, while another plate was incubated at 37 °C with blue light irradiation. The fluorescence signal of GFP and cell density (OD_600_) were measured at various time points during induction using a multimode plate reader (Infinite Pro200, Tecan). The blue light (460 nm) condition was achieved by placing a LED panel (270 × 170 × 1.6 mm, 120 W) over the agar plate or 24-well plates, adjusting the voltage intensity (0–10 V) with a controller to regulate the light intensity. The light intensity was measured with a Quantum meter (Apogee Instruments, model MQ-510). For GFP fluorescence signal measurement, the multimode plate reader (Infinite Pro200, Tecan) with an excitation wavelength of 488 nm and an emission wavelength of 530 nm was utilized.

### Screening of light-responsive LacI variants

BL21(DE3) was used for the screening of light-responsive LacI variants. Plasmids, including pZH37, pZH35 and its derivatives harboring different candidate LacI variants, were individually transformed into BL21(DE3) competent cells. A single colony was picked cultured for overnight at 37 °C. Subsequently, the cultures were inoculated into two 24-well plates (NEST, 702001, China), each well containing 1 ml LB medium containing kanamycin (50 μg/ml). One plate was exposed to blue light (60 μmol m^−2^s^−1^) and cultured for 12 h at 37 °C, while another one was cultured in the dark. At the end of 12-h incubation, the GFP fluorescence and optical density (OD_600_) of each sample were measured.

### Characterization of OptoLacI variants and Opto*E.coli* system

To optimize the induction intensity of the Opto*E.coli*^Dark^ system, we transformed OptoBL21^Dark^ competent cells with serial plasmids carrying LacI^K84^ saturation mutations. For the subsequent strain growth and GFP fluorescence measurement of the colonies from the plates, please refer to the “Growth and measurement of bacterial cultures” section.

To investigate the impact of *lacO1* tandem number on the induction effect of the Opto*E.coli* system, blue light-induced gene expression plasmids (pZH251, pZH261, pZH58, and pZH262) were respectively transformed into the OptoBL21^Light^ strains for the Opto*E.coli*^Light^ system. Freshly transformed cells were incubated overnight in the dark. For the Opto*E.coli*^Dark^ system, dark-induced gene expression plasmids (pZH91, pZH96, pZH36, pZH99, pML290, pML291, pZH151, pML308, pML310, and pML313) were separately transformed into the OptoBL21^Dark^ competent cells. Freshly transformed cells were incubated overnight under blue light condition (40 μmol m^−2^s^−1^). For the subsequent strain growth and GFP fluorescence measurement methods, please refer to the “Growth and measurement of bacterial cultures” section.

To assess the influence of different intensities of blue light on the Opto*E.coli*^Light^ system, a freshly transformed single colony (the OptoBL21^Light^ strain + pZH251) was selected and cultured in the dark condition for 12 h. The cells were then inoculated into 24-well plates. The 24-well plates designated for induction were subjected to blue light, intensities ranging from 10 to 100 μmol m^−2^s^−1^, for 10 h at 37 °C to stimulate GFP expression. The control plate was incubated for 10 h in dark (0 μmol m^−2^s^−1^). For the Opto*E.coli*^Dark^ system, a freshly transformed single colony (the OptoBL21^Dark^ strain + pML308) was selected and cultured, the dark-induced 24-well plates were kept in darkness for 12 h at 37 °C for induction, while the control plate was exposed to blue light, intensities ranging from 60 to 120 μmol m^−2^s^−1^. For the subsequent GFP fluorescence measurement methods, please refer to the “Growth and measurement of bacterial cultures” section.

To characterize the impact of various blue light pulse modes on the tunability of the Opto*E.coli* system, a freshly transformed single colony (the OptoBL21^Light^ strain + pZH251 for the Opto*E.coli*^Light^ system, or the OptoBL21^Dark^ strain + pML308 for the Opto*E.coli*^Dark^ system) was cultured in the dark or in blue light (60 μmol m^−2^s^−1^) overnight. Cells were inoculated into six separate 24-well plates and incubated at 37°C for 12 h, employing different pulse modes: full blue light, 1 s off / 1000 s on, 10 s off / 1000 s on, 100 s off / 1000 s on, and full darkness. For the subsequent GFP fluorescence measurement, please refer to the “Growth and measurement of bacterial cultures” section.

#### Characterization of the tunability of the Opto*E.coli* system under different induction starting time

For the Opto*E.coli*^Light^ system, a freshly transformed single colony (the OptoBL21^Light^ strain + pZH251) was selected and cultured overnight. The culture was subcultured in six 24-well plates until the cell density OD_600_ reached to 0.09, 0.38, 0.56, 0.65, and 0.81, respectively. The culture was then induced with blue light (60 μmol m^−2^s^−1^), while the control 24-well plate remained in the dark for 12 h. For the Opto*E.coli*^Dark^ system, the overnight culture (the OptoBL21^Dark^ strain + pML308) was subcultured in six 24-well plates until the cell density OD_600_ reached to 0.18, 0.28, 0.54, 0.75, and 1.14, respectively. Subsequently, the cultures were moved into the dark condition, while the control plate was exposed to blue light irradiation (60 μmol m^−2^s^−1^) for 12 h. For the subsequent GFP fluorescence measurement, please refer to the “Growth and measurement of bacterial cultures” section.

### Preparation of bacterial photographs with the Opto*E.coli*^Light^ system and the Opto*E.coli*^Dark^ system

To evaluate the spatial control performance of the Opto*E.coli*^Light^ system, a freshly transformed single colony (the OptoBL21^Light^ strain + pZH251) was cultured overnight under dark condition. 200 μl of 1:200 diluted overnight-culture was spread onto a 9 cm LB agar plate followed by incubation in the dark for 6–8 h. Subsequently, an aluminum foil with a triangular frame was utilized to cover the top of the plate, exposing a triangular region to blue light (40 μmol m^−2^s^−1^) for 48 h to induce GFP expression. For the Opto*E.coli*^Dark^ system, a single colony (the OptoBL21^Dark^ strain + pML308) was incubated overnight under blue light (60 μmol m^−2^s^−1^). 200 μl of 1:200 diluted overnight-culture was spread onto a 9 cm LB agar plate. The plate was covered with a translucent triangular aluminum foil, leaving the remaining areas exposed to blue light (40 μmol m^−2^s^−1^). GFP fluorescence imaging was conducted using the Bio-Rad ChemiDoc MP imaging system, and ImageJ software was utilized for image processing (27).

### Protein production

To assess protein production using the Opto*E.coli*^Dark^ system in comparison to the IPTG-induced gene expression system, we introduced the plasmids pML333 and pML351 into OptoBL21^Dark-2^, and the plasmid pML353 into OptoBL21^Dark^. Single colonies were incubated overnight at 37 °C under blue light (60 μmol m^−2^s^−1^). The cells were then inoculated into 24-well plates and incubated at 37 °C under blue light (120 μmol m^−2^s^−1^) until the OD_600_ reached 0.1 (OptoBL21^Dark-2^+ pML333, OptoBL21^Dark^+ pML353), 0.8 (OptoBL21^Dark-2^+ pML351), and 0.2 (OptoBL21^Light^+ pML409, OptoBL21^Light^+ pML410), respectively. The 24-well plates were then transferred to the dark condition or blue light condition (40 μmol m^−2^s^−1^) to induce the expression of target genes. At different time points, 200 μl of cells were collected and centrifuged at 12000 rpm to remove the culture medium. The cells were resuspended in 100 μl of PBS. 15 μl cells were taken to prepare protein samples for further SDS-PAGE electrophoresis. SDS-PAGE gels were imaged on a Bio-Rad ChemiDoc MP imaging system.

### Preparation and analytical methods for 1,3-PDO fermentation samples

Plasmid pJH10 was transformed into BL21(DE3) competent cells to construct the IPTG-induced 1,3-PDO synthesis strain, while plasmid pJH11 was transformed into OptoBL21^Dark^ competent cells to construct the dark-induced 1,3-PDO synthesis strain. For the IPTG-induced 1,3-PDO synthesis strain, single colony was selected and cultured in LB medium containing streptomycin (50 μg/ml) at 37 °C for 12–18 h. The culture was then sub-cultured into two 24-well plates and grown in the dark at 37 °C until reaching an OD_600_ of 0.1–0.6. One plate's culture was supplemented with 10 g/l CaCO_3_ and 8 mg/l VB_12_ and induced with 1 mM IPTG to promote 1,3-PDO synthesis, while the cultures in other plate were not induced. Samples were collected after 48 h of fermentation at 30 °C. For the dark-induced 1,3-PDO synthesis strain (OptoBL21^Dark^+ pJH11), single colony was selected and cultured in LB medium containing streptomycin at 37 °C for 12–18 h. Cells were then sub-cultured into two 24-well plates and grown at 37 °C under blue light (80 μmol m^−2^s^−1^) until the OD_600_ reached 0.1–0.6. CaCO_3_ and VB_12_ were then supplemented to a final concentration of 10 g/l and 8 mg/l, respectively, to modulate the pH and promote the 1,3-PDO biosynthesis according to the previous research (28). One of the 24-well plates was kept in the dark, while the other plate was exposed to blue light (80 μmol m^−2^s^−1^) during fermentation. Samples were collected after 48 h of fermentation at 30 °C.

At the end of fermentation, 800 μl of the culture supernatant was centrifuged at 12000 rpm for 10 min to collect the clarified lysate, and 1,3-PDO concentration was measured using a Waters HPLC system equipped with a refractive index detector, and an organic acid analytical column (Aminex HPX-87H, 300 mm × 7.8 mm, 9 μm; Bio-Rad) was used for separation. The column was eluted with 5 mM sulfuric acid at a flow rate of 0.6 ml/min at 55°C.

### Preparation and analytical methods for EGT fermentation samples

Plasmid pYJ111 was transformed into BL21(DE3) competent cells to construct the IPTG-induced EGT synthesis strain. Plasmid pYJ165 was transformed into OptoBL21^Dark^ competent cells to construct the dark-induced EGT synthesis strain. Single colony was selected and cultured overnight at 37 °C in 10 ml of LB liquid medium supplemented with kanamycin (50 μg/ml). The next day, 10 μl of the overnight cultures were used to inoculate 1 ml of fermentation medium in 24-well plates. The IPTG-induced EGT synthesis strain (BL21 (DE3) + pYJ111) was grown at 37 °C until the OD_600_ reached 5.0, followed by the addition of IPTG to a final concentration of 1 mM. No IPTG was added in the control group. The cells were further cultured for 48 h at 28 °C. The dark-induced EGT synthesis strain (OptoBL21^Dark^+ pYJ165) was cultured at 37 °C under blue light irradiation (60–70 μmol m^−2^ s^−1^) until the OD_600_ reached 5.0. The cells were then transferred to the dark and further cultured for 48 h at 28 °C. The fermentation medium was composed of the following ingredients (w/v): 2.4% yeast extract, 1.2% tryptone, 0.3% NaCl, 0.2% K_2_HPO_4_, 0.05% MgSO_4_·7H_2_O, 3% glycerol, 0.006% ammonium ferric citrate (AFC), 0.1% anhydrous l-cysteine hydrochloride, 0.1% l-histidine, and 0.1% l-methionine.

At the end of fermentation, the fermentation broth from each well was collected and subjected to incubation at 100 °C for 10 minutes. Subsequently, the samples were centrifuged at 12 000 rpm for 5 min. For further purification, the samples were then filtered through a 0.22 μm membrane filter prior to analysis by HPLC according to the previous research (29). The HPLC system used an amino column (Zorbax NH2, 4.6 × 250 mm, 5 μm, Agilent). The mobile phase consisted of a mixture of 80% acetonitrile and 20% water, at a flow rate of 1.0 ml/min. 20 μl of samples were used for injection into the HPLC system. The detection wavelength and column temperature were set at 254 nm and 35 °C, respectively.

### Expression, purification and size exclusion chromatography of OptoLacI

#### Expression of the OptoLacI^D2^ and the OptoLacI^L^

The OptoLacI^D2^ gene was inserted into the dark-induced expression vector (carrying 4× *lacO1*) by Gibson assembly to obtain the expression plasmid pML363. Similarly, the OptoLacI^L^ gene was inserted into the blue light inducible expression vector (carrying 3× *lacO1*) by Gibson assembly to obtain the expression plasmid pML364. The C-terminal of OptoLacI^D2^ and OptoLacI^L^ were fused with a 6× His tag. Plasmids pML363 and pML364 were transformed into BL21 (DE3) cells. Single colony was selected and transferred to 10 ml LB liquid medium supplemented with kanamycin (50 μg/ml). The culture was incubated at 37 °C overnight. The culture was then transferred to 800 ml LB liquid medium and incubated at 37 °C until the OD_600_ reached 0.6–0.8. The culture was then cooled to 16 °C. A final concentration of 0.4 mM IPTG was added to induce gene expression and the culture was incubated for an additional 18 h. Cells were harvested by centrifugation at 5000 rpm for 15 min, followed by resuspension in buffer A (20 mM Hepes, pH 7.5, 500 mM NaCl).

#### Purification and size exclusion chromatography of OptoLacI

Harvested 2 × 800 ml cells for ultrasonic disruption (50 Hz, 1 s on, 3 s off, 40 min). The cell lysate was centrifuged at 12 000 rpm for 1 hour and the precipitate was discarded. DNAse I was added to the supernatant at a final concentration of 0.1 mg/ml and incubated at 30 °C for 1.5 h. Centrifuge again at 12 000 rpm for 1 h. Two Ni-NTA affinity chromatography (using a HisTrap FF column, 5 ml, Cytiva) followed by size exclusion chromatography (using a Superdex 200 Increase 10/300 GL column, Cytiva) was performed to obtain purified OptoLacI^D2^ and OptoLacI^L^. For Ni-NTA affinity chromatography, buffer B (20 mM Hepes, pH 7.5, 200 mM NaCl) and buffer C (20 mM Hepes, pH 7.5, 200 mM NaCl, 600 mM imidazole) were used. For size exclusion chromatography, buffer B (20 mM Hepes, pH 7.5, 200 mM NaCl) was used. Purified target proteins were then incubated under dark or blue light illumination for 1 hour, or pre-incubated OptoLacI^L^ with *lacO1* (10 μM, 300 μl) for 1 hour at 4 °C, followed by further size exclusion chromatography under dark and blue light illumination conditions.

## Results

### Design, construction and screening of OptoLacI

**Figure 1.**
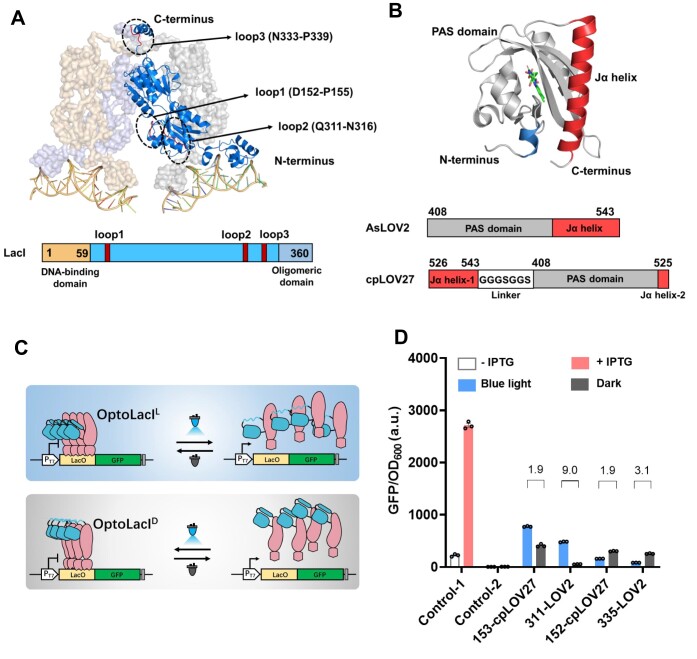
Design and construction of the light-controlled repressor OptoLacI. (**A**) The design of the insertion sites of the photosensitive domain based on the structure of LacI (PDB ID:1LBG). Loop1 (D152-P155) and loop2 (Q311-N316) are in close proximity to the DNA-binding domain of LacI^W220F^, exerting an influence on the binding interaction between LacI and the operator. Loop3 (N333-P339) is positioned close to the oligomeric domain, affecting the oligomerization state of LacI. (**B**) The photosensitive domains used in this study. AsLOV2 domain contains the PAS core domain and the Ja helix at the N-terminus (PDB ID: 2V1A). cpLOV27 is a circular arrangement mutant of AsLOV2. The N-terminus of AsLOV2 is connected to the C-terminus via the GGGSGGS linker and is interrupted between the 525th and 526th residues (E525 and R526). (**C**) Schematic diagrams of the mechanisms for inducing target gene expression using OptoLacI^L^ under blue light (top) and OptoLacI^D^ under dark condition (bottom), respectively. (**D**) OD_600_-normalized GFP fluorescence intensity of BL21(DE3) carrying wild type LacI (Control-1) induced by IPTG. OD_600_-normalized GFP fluorescence intensity of BL21(DE3) carrying variant LacI^W220F^ (Control-2), and OptoLacI variants (LacI^W220F, 153-cpLOV27^, LacI ^W220F, 311-LOV2^, LacI ^W220F, 152-cpLOV27^ and LacI^W220F, 335-LOV2^, respectively) under blue light and dark conditions. Open circles represent individual data points. Error bars represent the standard deviation of at least three biological replicates.

To achieve direct optical control of the transcriptional regulator LacI, we hypothesized that insertion of a photosensory module into LacI could allosterically alter the conformation of its DNA binding surface, thereby disrupting DNA recognition and binding through light-induced conformational changes. The N-terminal domain of LacI is responsible for its specific binding to operator DNA, while the C-terminal domain plays a critical role in facilitating the dimerization and tetramerization of LacI (Figure 1A) (4,30). For the insertion regions, we selected two solvent-exposed loops, namely loop1 (D152-P155) and loop2 (Q311-N316), in the N-terminal domain, and one solvent-exposed loop, namely loop3 (N333-P339), in the C-terminal domain (Figure 1A). The LOV2 domain derived from *Avena sativa* phototropin 1 (AsLOV2) and its circular permutant (cpLOV27) were utilized as the light-responsive modules (Figure 1B) (20,31). To screen and evaluate the light-responsive LacI variants (OptoLacI) (Figure 1C), we made modifications to the pET-28a plasmid. This involved replacing the native LacI with a mutant (LacI^W220F^) that exhibits an increased repression dynamic range and reduced leakiness (32). Additionally, we introduced two extra copies of the tandem lac operator (*lacO*) sequence and employed green fluorescent protein (GFP) as the reporter. By inserting LOV2 or cpLOV27 into the solvent-exposed loops of LacI^W220F^, we constructed seventeen LacI-LOV2 chimeras. Subsequently, these chimeras were individually transformed into *E. coli* BL21(DE3) for screening (Supplementary Figure S1A).

The BL21(DE3) strains carrying various chimeras were cultured under dark and blue light conditions, respectively. Strains harboring variants LacI^W220F, 153-cpLOV27^ and LacI^W220F, 311-LOV2^ notably exhibited significantly higher levels of GFP expression, with a 1.9-fold and 9.0-fold increase, respectively, under blue light compared to dark conditions. Conversely, the strains expressing LacI^W220F, 152-cpLOV27^ and LacI^W220F, 335-LOV2^ variants showed higher GFP expression levels, with a 1.9-fold and 3.1-fold increase, respectively, in the dark compared to blue light. In contrast, the control strain carrying LacI^W220F^ did not respond to either darkness or blue light exposure, resulting in a lack of GFP expression (Figure 1D). To enhance the dynamic range and maximum expression level, we selected the two variants LacI^W220F, 311-LOV2^ and LacI^W220F, 335-LOV2^ for further optimization. These two light-controllable LacI variants were renamed as OptoLacI^L^ and OptoLacI^D^, respectively. Additionally, we replaced the two copies of the *LacI* gene present in the chromosomes of *E. coli* BL21 (DE3) with OptoLacI^L^ and OptoLacI^D^, respectively, resulting in the generation of two novel *E. coli* strains: OptoBL21^Light^ and OptoBL21^Dark^ (Supplementary Figure S1B and Supplementary Table S4). OptoBL21^Light^ strain, along with OptoLacI^L^ mutant carried on the expression vector plasmid, was utilized to establish the Opto*E.coli*^Light^ system. Similarly, OptoBL21^Dark^, combined with the expression vector containing OptoLacI^D^ mutant, was employed to construct the Opto*E.coli*^Dark^ system.

### Optimization and characterization of the Opto*E.coli*^Light^ system

**Figure 2.**
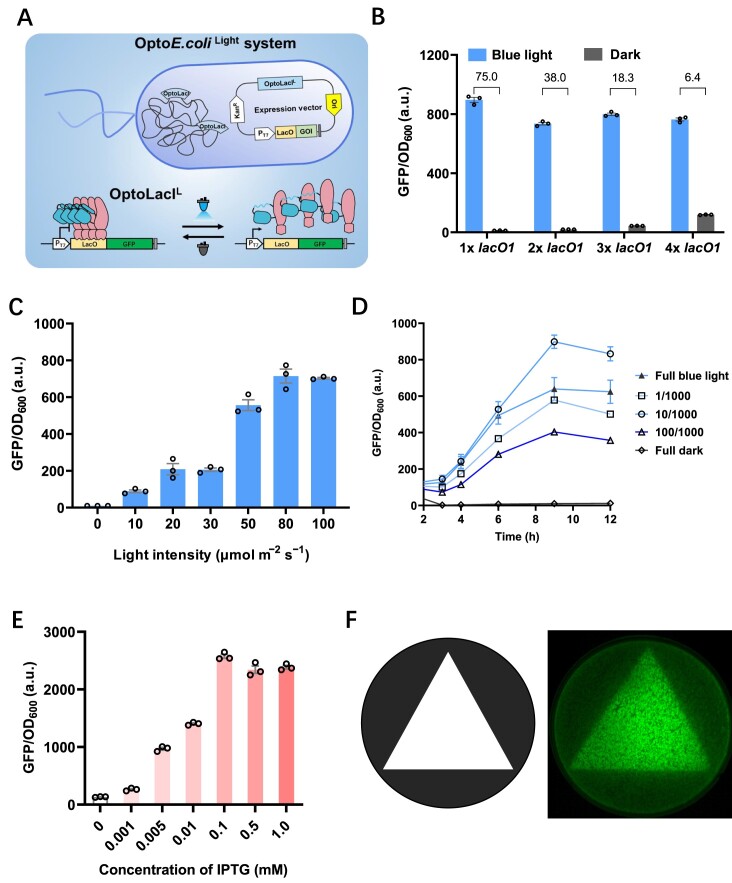
The Opto*E.coli*^Light^ system excels in tunability, induced rigidity, and spatial rigidity. (**A**) Schematic diagrams of the mechanism of the Opto*E.coli*^Light^ system. (**B**) Blue light induced GFP fluorescence of OptoBL21^Light^ strain transformed with plasmids carrying different numbers of operator *lacO1*. (**C**) Blue light intensity-dependent induction of GFP expression by the Opto*E.coli*^Light^ system. (**D**) GFP fluorescence intensity induced by various blue light pulses in the Opto*E.coli*^Light^ system. The blue light pulse modes: full blue light exposure, 1 s off / 1000 s on, 10 s off /1000 s on, 100 s off /1000 s on, and full darkness. (**E**) Comparison of GFP fluorescence intensity induced by different IPTG doses (0, 0.001, 0.005, 0.01, 0.1, 0.5, and 1.0 mM) in a conventional BL21(DE3) strain with an expression vector (pET-28a). (**F**) The Opto*E.coli*^Light^ system enables the production of high-contrast *E. coli* graphics. The triangular area used to expose to blue light (left). A triangular pattern generated by blue light induced GFP expression (right). The GFP fluorescence intensity was normalized to OD_600_. Open circles represent individual data points. Error bars represent the standard deviation of at least three biological replicates.

The Opto*E.coli*^Light^ system using blue light to induce the gene expression (Figure 2A). To further optimize this system, we investigated the impact of the copy number of operator *lacO1* on its overall performance and functionality utilize the OptoBL21^Light^ strain. The dynamic range in gene expression of the Opto*E.coli*^Light^ system, under light and dark conditions, showed a tendency to decrease from 75-fold to 6.4-fold with an increasing copy number of *lacO1*. The highest induction fold, reaching up to 75-fold, was achieved when the expression plasmid contained one *lacO1*, and the induced expression intensity reached its maximum (Figure 2B and Supplementary Figure S2A). Accordingly, induction leakage gradually increased with the addition of *lacO1* copies. Thus, we chose to use a single copy of *lacO1* in the Opto*E.coli*^Light^ system to optimize and characterize its performance.

To further characterize the blue light control performance of the Opto*E.coli*^Light^ system, we utilized the OptoBL21^Light^ strain with the largest dynamic range and highest expression of blue light induction as the subject of the study. Firstly, we examined the effect of blue light intensity, ranging from 0 to 100 μmol m^−2^s^−1^, on the Opto*E.coli*^Light^ system. The results exhibited a gradual increase in GFP expression as the intensity of blue light increased, with the GFP expression level reached its peak expression at an intensity of 80 μmol m^−2^s^−1^ (Figure 2C). Moreover, minimal leakage of GFP expression was observed in the absence of light (0 μmol m^−2^s^−1^), highlighting the high specificity of this blue light-induced system.

Subsequently, we explored the tunability of the Opto*E.coli*^Light^ system by employing pulsed light to induce gene expression. The pulse modes encompassed five conditions: full blue light, 1 s off /1000 s on, 10 s off /1000 s on, 100 s off /1000 s on, and full darkness, with induction for a duration of 12 h. The expression of GFP rapidly increased from 3 to 9 h of induction, reaching its maximum at 9 h across all conditions. As we expected, the pulse pattern of 10 s off /1000 s on induced the highest GFP expression, followed by the other blue light pulse conditions, decreasing in the order of full blue light, 1 s off / 1000 s on, and 100 s off /1000 s on. Almost no GFP expression was observed under full darkness (Figure 2D). These results indicate the remarkable tunability of Opto*E.coli*^Light^ system, allowing customization of the target protein's expression level. Moreover, the absence of expression leakage under complete darkness conditions demonstrates the system's stringent control. Moreover, the Opto*E.coli*^Light^ system exhibits tunable properties similar to the IPTG-induced gene expression system, with the difference that the tunability of the former is achieved by precise control of the blue light dose, whereas the latter is achieved by adjustment of the IPTG concentration (Figure 2E).

To assess the impact of the induction starting time on the Opto*E.coli*^Light^ system, cells were incubated under dark condition until the cell density OD_600_ reached 0.09, 0.38, 0.56, 0.65, and 0.81, respectively. Subsequently, blue light irradiation (100 μmol m^−2^s^−1^) was initiated to induce GFP expression. The results indicated that GFP expression remained consistent among cells with different initial induction densities when induced with blue light for 6 h. However, variations in GFP expression became noticeable between strains with different initial induction times after 6–12 h of blue light induction (Supplementary Figure S2B). These findings suggest that slight adjustments in blue light-induced gene expression can be achieved by modifying the onset time of the induction when utilizing the Opto*E.coli*^Light^ system. Additionally, as part of the comprehensive characterization of the Opto*E.coli*^Light^ system, we proceeded to evaluate its spatial rigidity. To achieve this, we coated the agar plate with aluminum foil leaving a triangular region exposed on the top surface of the plate. Subsequently, we induced GFP expression using blue light specifically within this triangular region. The Opto*E.coli*^Light^ system exhibited a highly sensitive light response, resulting in well-defined patterned edges with exceptional contrast (Figure 2F). This enabled the capture of high-quality photographs showcasing the bacteria on agar plate.

### Optimization and characterization of the Opto*E.coli*^Dark^ system

**Figure 3.**
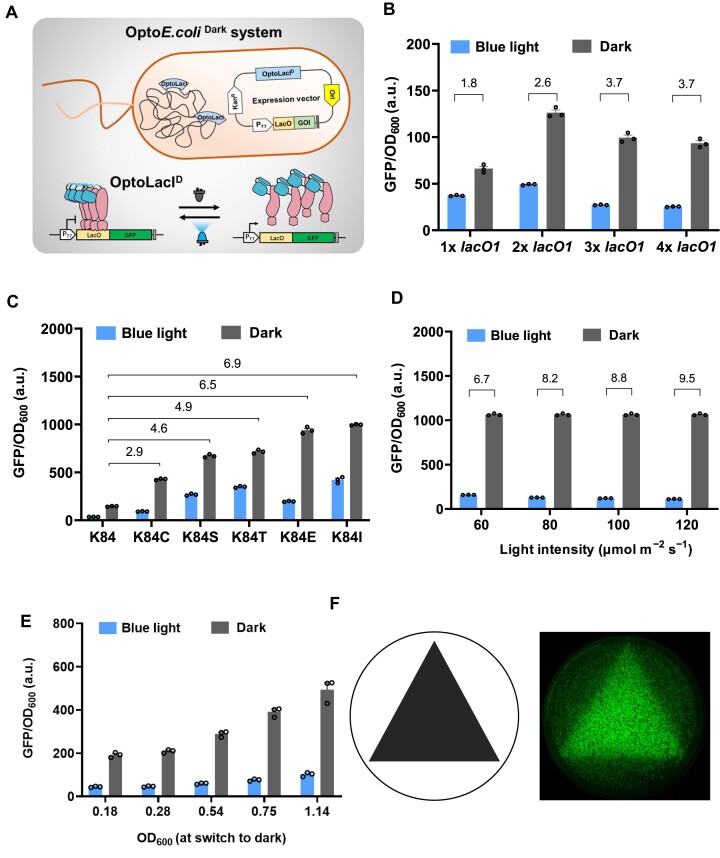
The Opto*E.coli*^Dark^ system exhibited the advantages in tunability, induced rigidity, and spatial rigidity. (**A**) The schematic diagrams of the mechanism of the Opto*E.coli*^Dark^ system. (**B**) Optimization of the number of operators *lacO1* in the Opto*E.coli*^Dark^ system. (**C**) Substituting Lys84 with cysteine (C), serine (S), threonine (T), glutamate (E) or isoleucine (I) significantly enhanced GFP expression in the Opto*E.coli*^Dark^ system under dark condition. (**D**) Blue light intensity-dependent induction of GFP leakage expression by the Opto*E.coli*^Dark^ system. (**E**) The difference in GFP fluorescence intensity between the different OD_600_ was particularly significant within the first 4 h. The GFP fluorescence intensity was normalized to OD_600_. Open circles represent individual data points. Error bars represent the standard deviation of at least three biological replicates. (**F**) The Opto*E.coli*^Dark^ system allows the production of high-contrast *E. coli* graphics. The triangular photomask used to produce the *E. coli* picture (left), the triangular pattern generated by dark-induced GFP expression for 24 h (right).

The Opto*E.coli*^Dark^ system induces gene expression under dark condition, presenting practical advantages for industrial-scale applications (Figure 3A). We assessed the impact of the copy number of operator *lacO1* in the Opto*E.coli*^Dark^ system with the OptoBL21^Dark^ strain. GFP expression in both strains reached the maximum after 12 h of dark induction (Supplementary Figure S3). The dynamic range in gene expression within the Opto*E.coli*^Dark^ system increased as the copy number of *lacO1* rose up to four. The OptoBL21^Dark^ strain showed a maximum dark induction range of 3.7-fold (Figure 3B). To further intensify the induction in the Opto*E.coli*^Dark^ system, we modified the tetramer assembly of OptoLacI^D^, specifically focusing on the lysine (K) residue at position 84 of LacI protein, which plays a crucial role in the tetramer assembly (33,34). Important observations were made through site-saturation mutation of Lys84 (K84) and assessing the induction effects of the mutants in OptoBL21^Dark^ strain. The results demonstrated a gradual increase in GFP expression under dark condition when Lys84 was replaced with cysteine (C), serine (S), threonine (T), glutamic acid (E), or isoleucine (I) (Figure 3C). Among these mutations, the substitution of lysine with isoleucine (K84I) resulted in the most substantial increase in dark-induced GFP expression, showing a remarkable 6.9-fold induction intensity compared to K84. Similarly, when lysine was replaced with glutamate (K84E), the intensity of dark-induced GFP expression increased by approximately 6.5-fold. Apparently, the dark-induced leakage of OptoLacI^D^-K84E was lower than that of OptoLacI^D^-K84I, even though both mutants exhibited similar dark-induced GFP expression. Therefore, we designated the mutant with K84E substitution in OptoLacI^D^ as OptoLacI^D2^. Furthermore, substituting K84 with other amino acids did not result in a noteworthy increase in dark-induced GFP expression for the mutants (Supplementary Figure S4A).

To further investigate the leakage of the Opto*E.coli*^Dark^ system, we constructed a set of plasmids with varying copy numbers of *lacO1*, ranging from 1 to 8. As expected, the dynamic range of the Opto*E.coli*^Dark^ system exhibited a gradual increase with the growing number of *lacO1* copies, reaching a peak at four copies with a 7.2-fold induction (Supplementary Figure S4B). Additionally, a new *E. coli* strain, OptoBL21^Dark-2^, was developed by replacing the two copies of the LacI gene present in the chromosomes of *E. coli* BL21 (DE3) with the OptoLacI^D2^ mutant. We then established the Opto*E.coli*^Dark-2^ system by introducing the OptoLacI^D2^ mutant on an expression vector plasmid in this strain (Supplementary Figure S4C). However, our comparison of the dark induction effect between the Opto*E.coli*^Dark-2^ system and the original Opto*E.coli*^Dark^ system revealed that the former did not show superiority in terms of induction intensity and dynamic range (Supplementary Figure S4D). Subsequently, we investigated the tunability of the Opto*E.coli*^Dark^ system by subjecting it to different intensities of blue light, ranging from 60 to 120 μmol m^−2^ s^−1^. Additionally, we examined the leakage expression of the system at these different blue light intensities. The results demonstrated that as the intensity of blue light increased, the leakage expression of the Opto*E.coli*^Dark^ system exhibited a gradual decrease (Figure 3D). These observations suggested that by appropriately increasing the intensity of blue light exposure, undesired leakage in the Opto*E.coli*^Dark^ system can be effectively minimized.

To assess the effect of when to start induction in the Opto*E.coli*^Dark^ system, we incubated the cells under blue light condition until they reached different cell densities (OD_600_, ranging from 0.18 to 1.14), at which we switched them from blue light to dark condition to initiate the expression. Our observations revealed that the rate of increase in GFP expression induced by darkness was higher when the OD_600_ of the strain was relatively larger throughout the time course (Supplementary Figure S5A). Moreover, a higher OD_600_ resulted in a greater amount of GFP expression by the strain during the same induction time (Figure 3E). These findings indicate that the rate of dark-induced gene expression can be effectively adjusted by appropriately modulating the induction start time when utilizing the Opto*E.coli*^Dark^ system.

In addition, we explored the tunability of the Opto*E.coli*^Dark^ system by utilizing pulsed light to induce gene expression. We tested five pulse modes, which included full darkness, 1 s on /1000 s off, 10 s on /1000 s off, 100 s on /1000 s off, and full blue light. The highest GFP expression was induced by full darkness and the lowest GFP expression was observed with the 100 s on /1000 s off pulse mode, and continuous blue light exposure failed to induce GFP expression (Supplementary Figure S5B). These findings demonstrate that the Opto*E.coli*^Dark^ system can be effectively controlled using different pulse modes of blue light. This flexibility is one of the advantages of the system, offering diverse options for precise control of gene expression.Another advantage of light-controlled gene expression is spatial controllability, where the system can precisely regulate gene expression in specific regions. To comprehensively assess the spatial rigidity of the Opto*E.coli*^Dark^ system, we coated the surface of an agar plate with a triangular aluminum foil, creating distinct triangular region on the plate. This allowed us to induce GFP expression using darkness specifically within the triangular area. The Opto*E.coli*^Dark^ system exhibited high spatial rigidity, effectively regulating gene expression within the designated triangular region (Figure 3F).

To date, we have successfully developed two light-controlled *E. coli* gene expression systems: Opto*E.coli*^Light^ system and Opto*E.coli*^Dark^ system, both exhibiting excellent light control performance. In our evaluation, we compared these systems with the IPTG-induced gene expression system. Both the Opto*E.coli*^Light^ system and the Opto*E.coli*^Dark^ system exhibited lower GFP expression leakage than the IPTG-induced system. Although the level of GFP expression was lower than that achieved with IPTG, both light-controlled systems still displayed robust gene expression (Supplementary Figure S6A). Furthermore, the Opto*E.coli*^Light^ system and the Opto*E.coli*^Dark^ system present distinct advantages for gene expression. They offer precise spatial and expression level tunability, rendering them more attractive options for inducing gene expression. Additionally, these systems are cost effective, adding significant value for various biological applications.

### Application of the Opto*E.coli*^Dark^ system in protein production

**Figure 4.**
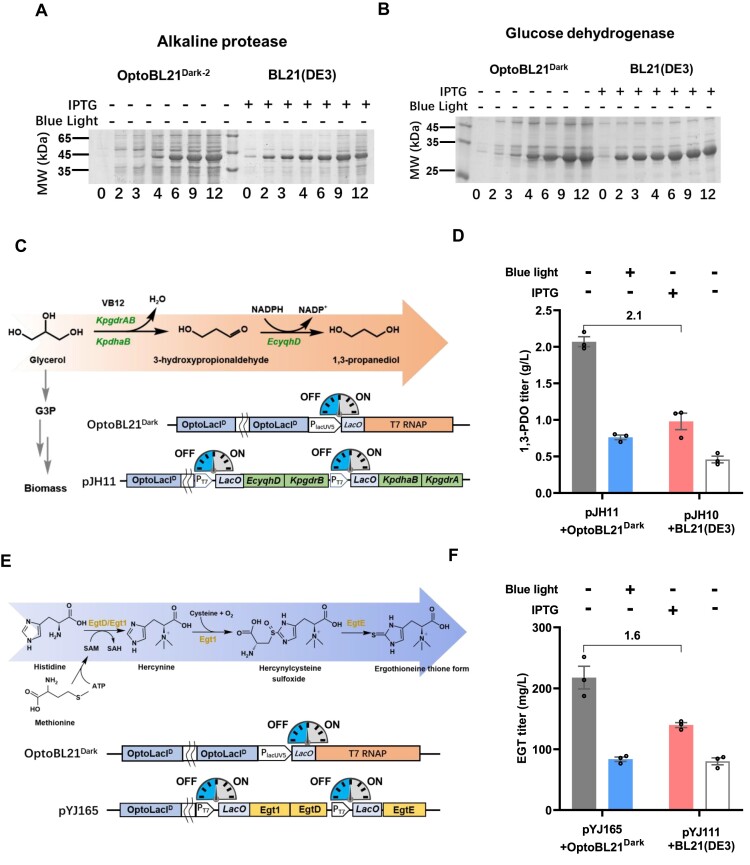
Application of the Opto*E.coli*^Dark^ system in protein production and control of metabolic pathways. (A, B) Production profiles of alkaline protease (**A**) and glucose dehydrogenase (**B**) using the Opto*E.coli*^Dark^ system. Inductions were initiated by switching the culture from blue light to darkness at their optimal cell density (OD_600_= 0.1). The IPTG-induced system served as a control, with induction triggered by adding 1 mM IPTG at the optimal cell density (OD_600_= 0.5). Samples were collected at 0, 2, 3, 4, 6, 9, and 12 h of induction to monitor target protein production. (**C**) Optogenetic control of the 1,3-propanediol (1,3-PDO) biosynthetic pathway using the Opto*E.coli*^Dark^ system. The OptoLacI^D^ protein regulates the expression of T7 RNA polymerase in the OptoBL21^Dark^ strain, along with glycerol dehydrogenase (*KpdhaB*), glycerol dehydrogenase reactivase (*KpgdrAB*), and alcohol dehydrogenase (*EcyqhD*) on the plasmid. (**D**) Comparison of the Opto*E.coli*^Dark^ system with IPTG induction system for 1,3-PDO production. (**E**) Optogenetic control of the ergothioneine (EGT) biosynthetic pathway using the Opto*E.coli*^Dark^ system. The OptoLacI^D^ protein controls the expression of T7 RNA polymerase in the OptoBL21^Dark^ strain, as well as S-adenosylmethionine-dependent methyltransferase (*EgtD*), C-S lyase (*EgtE*), and methyltransferase-sulfoxide synthase (*Egt1*) on the plasmid. (**F**) Comparison of the EGT production in the Opto*E.coli*^Dark^ system and the IPTG-induced system. Open circles represent individual data points. Error bars represent the standard deviation of at least three biological replicates.

The light-controlled gene expression systems provide a crucial cost-saving advantage by eliminating the need for expensive IPTG, making protein production more affordable and efficient. To validate the effectiveness of the Opto*E.coli*^Dark^ system for protein production, we constructed the genes for alkaline protease, PETase, and glucose dehydrogenase into a dark induction expression plasmid and pET-28a, respectively. In a comparison of protein production between the Opto*E.coli*^Dark^ system and the IPTG-induced expression system, Opto*E.coli*^Dark^ strains were incubated under blue light (80 μmol m^−2^ s^−1^) until the OD_600_ reached 0.1, followed by a switch to dark condition to induce expression. In contrast, the IPTG-induced strains were incubated until OD_600_ reached 0.5, and then 1 mM IPTG was added to induce gene expression. Notably, the Opto*E.coli*^Dark^ system exhibited minimal expression leakage during the growth phase when the gene expression was repressed (Figure 4A, B, time = 0 h). The production of alkaline protease increased rapidly after 4 h of dark induction, comparable to 9–12 h of IPTG induction (Figure 4A). Similarly, glucose dehydrogenase (GDH) expression increased significantly after 3 h of dark induction. After 9–12 h of dark induction, GDH expression levels reached a comparable magnitude to those observed with 9–12 h of IPTG induction (Figure 4B). Furthermore, PETase expression showed a substantial increase after 2 h of dark induction, surpassing the production achieved by IPTG-induced 9–12 h PETase production (Supplementary Figure S6B). Additionally, to explore the potential application of the Opto*E.coli*^Light^ system in protein production. We applied the Opto*E.coli*^Light^ system to the production of alkaline protease and glucose dehydrogenase (Supplementary Figures S6C, D). The results showed that for the same fermentation duration (12 h), the protein expression induced by blue light (at 40 μmol m^−2^s^−1^) was equivalent to that induced by IPTG (Figure 4A, B). These results indicate that both the Opto*E.coli*^Light^ system and the Opto*E.coli*^Dark^ system could efficiently produce target recombinant proteins under blue light and darkness, respectively, with expression levels comparable to those induced by IPTG.

### Application of the Opto*E.coli*^Dark^ system in controlling 1,3-propanediol production

In light of the promising prospects of optogenetics in metabolic flux control, we explored the application of the Opto*E.coli*^Dark^ system in the production of 1,3-propanediol (1,3-PDO) which is an important commodity chemical. A plasmid harboring an IPTG-induced 1,3-PDO biosynthesis pathway was constructed according to the previous research (35) and subsequently adapted to our Opto*E.coli*^Dark^ system and the IPTG-induced gene expression system, respectively (Figure 4C). The resultant strains control the 1,3-PDO production using light (OptoBL21^Dark^+ pJH11) or IPTG (BL21(DE3) + pJH10). For the dark-induced 1,3-PDO producing strain, the optical density at 600 nm (OD_600_) at which we switch fermentations from blue light to darkness has a significant impact on final 1,3-PDO titers (Supplementary Figure S7A). The 1,3-PDO titer achieved to 2.07 ± 0.12 g/l induced with dark at the optimal OD_600_ (OD_600_= 0.43), which is 110% higher than that of the fermentations induced with IPTG at their optimal OD_600_ of induction (0.98 ± 0.20 g/l) (Figure 4D and Supplementary Figure S7A, B). These results suggest that the Opto*E.coli*^Dark^ system holds promising applications in metabolic flux control and indicate that darkness can serve as a viable alternative to IPTG for inducing chemical production during *E. coli* fermentation.

### Application of the Opto*E.coli*^Dark^ system in controlling ergothioneine production

To assess the broad applicability of optogenetics in metabolic flux control, we conducted another investigation focused on harnessing the Opto*E.coli*^Dark^ system for the production of ergothioneine (EGT), a significant antioxidant compound derived from histidine (29,36,37) (Figure 4E). Similarly, an IPTG-induced EGT biosynthesis pathway was assembled according to the previous report (29) and thereafter modified and adapted to the Opto*E.coli*^Dark^ system and the IPTG-induced BL21(DE3) system, respectively (Figure 4E). These resulting strains exert control over biomass accumulation and EGT production, utilizing either blue light (OptoBL21^Dark^ + pYJ165) or IPTG (BL21(DE3) + pYJ111). The optical density at 600 nm (OD_600_) at which we switch fermentations from blue light to darkness has significant effect on final titers in the dark-induced system. The EGT titer achieved to 217.47 ± 18.63 mg/l when induced with dark at the optimal OD_600_ (OD_600_= 5.03), which is 60% higher than that of the fermentations induced with IPTG at their optimal OD_600_ of induction (139.61 ± 4.04 mg/l) (Figure 4F and Supplementary Figure S7C). This result further substantiates the efficacy of the Opto*E.coli*^Dark^ system in eliminating the requirement for costly chemical inducer IPTG by employing dark induction. Moreover, it offers supplementary evidence that underscores the advantages of the Opto*E.coli*^Dark^ system in enhancing chemical production in *E. coli*.

## Discussion

The blue light-sensitive LOV2 domain has been employed to engineer several proteins through fusion and insertion techniques, resulting in light-responsive properties that induce conformational and functional changes within the targeted proteins (21,38–40). Here, we demonstrate the successful engineering of the lactose repressor protein LacI into two light-controllable chimeras, namely OptoLacI^D^ and OptoLacI^L^. Specifically, OptoLacI^D^ functions as a repressor, binding to *lacO* and operating as a repressor in the presence of blue light, whereas OptoLacI^L^ exerts its repressive function in the dark. Leveraging the unique attributes of OptoLacI^L^ and OptoLacI^D^ proteins, we have successfully transformed *E. coli* BL21(DE3) and pET expression system into the Opto*E.coli*^Light^ and the Opto*E.coli*^Dark^ systems, offering a versatile range of applications in protein production and chemical biosynthesis.

Incorporating the LOV2 domain into the target protein to achieve light-driven conformational changes stands a direct and efficient strategy for optogenetic protein engineering. The OptoLacI^D^ and OptoLacI^L^ offer swift and direct light manipulation of their repressive functions. Additionally, as OptoLacI-based single-component optogenetic regulatory systems, the Opto*E.coli*^Dark^ and Opto*E.coli*^Light^ systems operate without the need for intricate signal transduction processes. The simplicity and direct control of these modules confer significant advantages over the OptoLAC system, which achieves optogenetic regulation by controlling LacI expression (14). The complexity of such circuits arises from the reliance on four proteins and three layers of logic gates to achieve light control over gene expression (Supplementary Table S5). Nevertheless, compared to the single-component eLightOn system (7), which has a high dynamic range (>500-fold). The dynamic range of our Opto*E.coli* system was limited (75-fold) and requires further optimization.

Supplying adequate light to large-scale reactors poses a substantial challenge in the practical application of optogenetic technology within the field of metabolic engineering. To address this issue in production applications, dark-induced optogenetic systems prove to be more suitable. In this context, microbial cell factories are cultivated under blue light to repress the expression of target genes and ensure biomass accumulation, followed by the production of the target product in the absence of blue light. Our Opto*E.coli*^Dark^ system along with previously reported dark-induced optogenetic systems, such as the EL222-GAL80 repressor-leveraged OptoINVRT circuit in yeast (8) and the pDawn system-based OptoLAC circuit in *E. coli* (14), have demonstrated the advantages of this strategy in the production of various proteins and chemicals. In contrast, the BLADE (blue light-inducible AraC dimers in *E. coli*) system is only capable of blue light-induced gene expression, potentially constraining its practical application (Supplementary Table S5). Additionally, the utilization of darkness as an inducer, in contrast to IPTG, does not cause toxicity to the cell. Consequently, the Opto*E.coli*^Dark^ system does not require the attainment of a high cell density at the start of induction. For instance, the optimal cell density (OD_600_) for the dark-induced production of alkaline protease and glucose dehydrogenase is 0.1. In large-scale fermentation processes, this allows for the cultivation of the seed culture to an OD_600_ of approximately 100 in a small transparent fermenter under blue light irradiation. Subsequently, the culture can be directly inoculated into a large fermentation tank (e.g. 1000 l or more), following the OD_600_= 0.1 criterion, for subsequent dark-induced fermentation. Furthermore, IPTG has the property of not being metabolized by cells (41), which leads to the difficulty of completely removing it from the product, and considering the safety of the product, IPTG induction has limitations in the production of food and pharmaceutical grade protein products. Conversely, dark induction is more suitable for the production of food and pharmaceutical grade protein products due to the absence of chemical residues and higher safety.

Tunability stands out as a prominent advantage of the opto-proteins and the optogenetic systems. The functions of optogenetic proteins can be rapidly activated or deactivated at the protein level. Through the application varying intensities and pulse patterns of blue light, the Opto*E.coli*^Dark^ and Opto*E.coli*^Light^ systems exhibit excellent spatiotemporal and dynamic regulation of gene expression (Figures 2, 3, Supplementary Figure S5). In contrast, chemical inducers cannot be removed once added to the reactor. Despite the advantages, the Opto*E.coli*^Dark^ and Opto*E.coli*^Light^ systems also have some drawbacks. Their maximum expression level of the target gene is still not high enough, lower than that induced by IPTG (Supplementary Figure S6A). This suggests that the Opto*-*systems are more suitable for applications not requiring high expression strength. However, given their high tunability, optimizing the blue light protocol may enhance the expression levels of the target genes. Moreover, we sought to investigate whether OptoLacI retained its responsiveness to IPTG. To address this inquiry, we have supplemented the IPTG induction experiments with Opto*E.coli*^Light^ and Opto*E.coli*^Dark^ systems. The results reveal that the Opto*E.coli* systems indeed remain responsive to IPTG (Supplementary Figure S7D), indicating that the incorporation of the LOV2 domain does not abolish the IPTG induction capability of OptoLacI. These findings underscore the versatility of the Opto*E.coli* system, as it accommodates various induction approaches tailored to specific application requirements.

The structural distinction between OptoLacI^D^ and OptoLacI^L^ lies in the location of the insertion of the LOV2 domain. The LOV2 domain in OptoLacI^L^ is positioned in loop2 (between residues 311–312), proximate to the *lacO*-binding region of LacI. In contrast, the LOV2 domain in OptoLacI^D^ is situated in loop3 (between residues 335–336) distant from the *lacO*-binding region and close to the α-helix responsible for mediating the oligomerization of LacI (Supplementary Figure S8). The crucial significance of the tetrameric structure of the LacI repressor in preserving the physiological function of the lactose repressor system has been documented in previous studies (4,42). Consequently, we posited that LOV2 influences the aggregation state of LacI monomer by undergoing conformational changes in response to blue light, thereby leading to the distinct functionalities of OptoLacI^D^ and OptoLacI^L^ upon exposure to blue light. Indeed, our investigation into the aggregation states of these two LacI variants aligns with this hypothesis that guided our initial design (Supplementary Figure S9). Blue light facilitates the aggregation of OptoLacI^D^ and the disaggregation of OptoLacI^L^, respetively. Conversely, under dark condition, OptoLacI^L^ exhibits aggregation while OptoLacI^D^ undergoes disaggregation, showcasing an opposing trend.

OptoLacI variants and the Opto*E.coli* systems provide a direct solution for optogenetic engineering of the chemical inducer-based transcription factors and gene expression systems. The substituting of chemical inducers with blue light or darkness offers advantages in reducing costs, minimizing pollution, and enabling more diverse and dynamic regulation in large-scale industrial fermentation productions. Additionally, the application of the LacI repressor has been extended to other bacteria beyond *E. coli* (43–45), as well as yeast (46) and mammalian cells (47,48). Hence, OptoLacI chimeras have the potential to be applied in a range of systems initially designed with LacI as a component, endowing them with the beneficial traits of optogenetics, including reversibility, spatiotemporal control and precise tunability.

## Supplementary Material

gkae479_Supplemental_File

## Data Availability

The data underlying this article are available in the article and in its online supplementary material.
