# Supplementary material for: OptoLacI: optogenetically engineered lactose operon repressor LacI responsive to light instead of IPTG
Source: Nucleic Acids Res. 2024 Jun 11;52(13):8003–16. doi: 10.1093/nar/gkae479 (PMC11260447; doi:10.1093/nar/gkae479)
Supplement: gkae479_Supplemental_File [file gkae479_supplemental_file.pdf]

Supplementary Data

Supplementary Data are available at NAR online.

Supplementary Figures

Supplementary Figure 1. Screening and optimization of the light-controlled repressor OptoLacI.

a, Screening for LacI variants with a blue light response was carried out in the BL21(DE3) strain under blue light and dark conditions. The control plasmid carryed the variants $LacI^{W220F}$, while the test plasmid included the variants $LacI^{W220F,\ 154\text{-}cpLOV27}$, $LacI^{W220F,\ 155\text{-}cpLOV27}$, $LacI^{W220F,\ 312\text{-}LOV2}$, $LacI^{W220F,\ 313\text{-}LOV2}$, $LacI^{W220F,\ 314\text{-}LOV2}$, $LacI^{W220F,\ 315\text{-}LOV2}$, $LacI^{W220F,\ 316\text{-}LOV2}$, $LacI^{W220F,\ 333\text{-}LOV2}$, $LacI^{W220F,\ 334\text{-}LOV2}$, $LacI^{W220F,\ 336\text{-}LOV2}$, $LacI^{W220F,\ 337\text{-}LOV2}$, $LacI^{W220F,\ 338\text{-}LOV2}$, $LacI^{W220F,\ 339\text{-}LOV2}$, respectively. **b,** Genomic composition of the BL21(DE3) strain, the $OptoBL21^{Dark}$ strain, and the $OptoBL21^{Light}$ strain in this study.

Supplementary Figure 2. Optimization and characterization of the Opto*E.coli*^{Light} system.

a, Blue light and dark-induced GFP fluorescence with the Opto*E.coli*^{Light} system ($OptoBL21^{Light}$ + expression plasmids carrying different copy numbers of *lacO1*). **b**, The effect of when to start induction in the Opto*E.coli*^{Light} system. Cells were incubated in the dark until they reached different cell densities (OD_{600}=0.09, 0.38, 0.56, 0.65, and 0.81), at which we switched them from dark to blue light condition to initiate the expression. The GFP fluorescence intensity was normalized to OD_{600}. Open circles represent individual data points. Error bars represent the standard deviation of at least three biological replicates.

Supplementary Figure 3. Time course of optimizing and characterizing the Opto*E.coli*^{Dark} system.

Time courses of dark and blue light-induced GFP fluorescence of the $OptoBL21^{Dark}$ strain transformed with plasmids carrying different copy numbers of *lacO1*. The GFP fluorescence intensity was normalized to OD_{600}. Open circles represent individual data points. Error bars represent the standard deviation of at least three biological replicates.

Supplementary Figure 4. Optimization of the induction intensity and the dynamic range of the Opto*E.coli*^{Dark} system.

a, Dark-induced GFP fluorescence of the $OptoBL21^{Dark}$ strain transformed with plasmids containing saturating mutants of $LacI^{K84}$. **b,** Optimization of the number of operators *lacO1* of the Opto*E.coli*^{Dark} system using the expression plasmids carrying the $LacI^{K84E}$ mutation. **c,** The schematic diagrams of the composition of the genomes of the $OptoBL21^{Dark\text{-}2}$ strain. The starting strain is BL21-Amp-Cm, created by replacing the two original LacI copies on the BL21(DE3) genome with ampicillin and chloramphenicol resistance genes, respectively. These two resistance genes were then replaced with the $OptoLacI^{D2}$ to obtain the $OptoBL21^{Dark\text{-}2}$ strain. **d,** Systematic comparison of the light control

performance of dark-induced GFP expression of the OptoBL21^{Dark-2} strain with that of the OptoBL21^{Dark} strain. Expression plasmids carrying 4x *lacO1* was transformed into the OptoBL21^{Dark-2} strain and the OptoBL21^{Dark} strain, respectively. The GFP fluorescence intensity was normalized to OD_{600}. Open circles represent individual data points. Error bars represent the standard deviation of at least three biological replicates.

Supplementary Figure 5. Characterization of the tunability of the Opto*E.coli*^{Dark} system.
a, Time courses of dark-induced GFP fluorescence in the OptoBL21^{Dark} strain carrying pML308 with different induction start time (OD_{600}=0.18, 0.28, 0.54, 0.75, and 1.14). **b**, Time courses of GFP fluorescence of the OptoBL21^{Dark} strain carrying pML308 with different blue light pulse modes (full blue light, 1 s on/ 1000 s off, 10 s on/ 1000 s off, 100 s on / 1000 s off, and full darkness). The GFP fluorescence intensity was normalized to OD_{600}. Error bars represent the standard deviation of at least three biological replicates.

Supplementary Figure 6. Comparison of light-controlled gene expression systems and IPTG induction system.
a, Comparison of GFP fluorescence of the dark-induced Opto*E.coli*^{Dark} system, the blue light-induced Opto*E.coli*^{Light} system, and the IPTG-induced system. The Opto*E.coli*^{Dark} system (OptoBL21^{Dark} strain+ pML308) was induced by darkness for 12 hours. The Opto*E.coli*^{Light} system (OptoBL21^{Light} strain+ pZH251) was induced by blue light (80 μmol m^{-2} s^{-1}) for 10 hours. The IPTG-induced system was induced by 1 mM IPTG for 12 hours. **b,** PETase production was carried out using the Opto*E.coli*^{Dark} system (OptoBL21^{Dark-2} strain + pML351) and induction was started by switching to darkness when the cell density OD_{600} reached 0.8. The IPTG-induced system was used as a control and induction was started by adding 1 mM IPTG when the cell density OD_{600} reached 0.5. The induction temperature for both systems were 37 °C. **c,** Alkaline protease production was carried out using the Opto*E.coli*^{Light} system (OptoBL21^{Light} strain + pML409). Induction was initiated by switching to blue light (40 μmol $m^{-2}s^{-1}$) when the cell density (OD_{600}) reached 0.2. **d,** Glucose dehydrogenase production was carried out using the Opto*E.coli*^{Light} system (OptoBL21^{Light} strain + pML410). Induction was initiated by switching to blue light (40 μmol $m^{-2}s^{-1}$) when the cell density (OD_{600}) reached 0.2.

Supplementary Figure 7. Optimization of induction timing in controlling metabolic pathways using the Opto*E.coli*^{Dark} system and the IPTG-induced system.
a, Optimization of the optimal induction timing for 1,3-PDO production using the Opto*E.coli*^{Dark} system. The fermentation broth was incubated under blue light (60 μmol m^{-2} s^{-1}) until the OD_{600} reached to 0.32, 0.43, 0.84, and 2.99, respectively. The fermentation was then transferred to the dark environment for further incubation. **b**, Optimization of the optimal induction timing for 1,3-PDO production using the IPTG-induced system. The fermentation broth was incubated until the OD_{600} reached to 0.31, 0.46, 1.05, and 3.18, respectively. Then, 1 mM IPTG was added to the fermentation

broth. **c,** Optimization of the optimal induction timing for EGT production using the Opto*E.coli*^{Dark} system. The fermentation broth was incubated under blue light (60 μmol m^{-2} s^{-1}) until the OD_{600} reached to 1.16, 2.71, 3.87, 5.03, and 6.97, respectively. The fermentation broth was then transferred to the dark environment for further incubation. Open circles represent individual data points. Error bars represent the standard deviation of at least three biological replicates. **d,** GFP fluorescence intensity resulting from IPTG induction was measured in both the Opto*E.coli*^{Dark} system and the Opto*E.coli*^{Light} system following exposure to 1 mM IPTG for a duration of 12 hours.

Supplementary Figure 8. Structural features of the OptoLacI^{L} and OptoLacI^{D2} proteins.
The structure of OptoLacI monomer (OptoLacI^{L} and OptoLacI^{D2}) comprises three major domains: a DNA binding domain (orange cartoon), a regulatory domain (green cartoon) that encompasses regions for oligomerization and for IPTG binding, and an inserted LOV2 domain (sky blue cartoon). **a,** The LOV2 domain (sky blue cartoon) is positioned in loop2 (between residues 311-312), proximate to the *lacO*-binding region of LacI. **b**, The LOV2 domain is situated in loop3 (between residues 335-336), distant from the *lacO*-binding region and in close proximity to the α-helix responsible for mediating the oligomerization of LacI. The structures of OptoLacI^{L} and OptoLacI^{D2} in **(a)** and **(b)** were predicted using the AlphaFold2 TIB Server (https://alphafold2.biodesign.ac.cn/). Images were generated using PyMOL software.

Supplementary Figure 9. Changes in the aggregation states of OptoLacI^{D2} and OptoLacI^{L} under dark and blue light conditions.
a, Size Exclusion Chromatography (SEC) analysis of OptoLacI^{D2} under dark (gray line) and blue light (blue line) conditions. **b,** Sodium Dodecyl Sulphate-Polyacrylamide Gel Electrophoresis (SDS-PAGE) analysis of samples collected at the peak positions of OptoLacI^{D2} in dark and blue light conditions in (**a**). **c,** SEC analysis of OptoLacI^{L} under dark (gray line) and blue light (blue line) conditions. **d,** SDS-PAGE analysis of samples collected at the peak positions of OptoLacI^{L} in dark and blue light conditions in (**c**). **e,** SEC analysis of an incubated mixture of OptoLacI^{L} with DNA (1x *lacO1*) on a Superdex 200 Increase 10/300 GL column under both dark and blue light conditions. **f,** SEC analysis of the DNA (1x *lacO1*) using a Superdex 200 Increase 10/300 GL column under dark condition. The orange line represents the peak plot of SEC using DNA under dark condition.

Supplementary Tables

Supplementary Table 1: Oligonucleotides used in this study.

Supplementary Table 2: Amino acid sequences used in this study.

Supplementary Table 3: Plasmids used in this study.

Supplementary Table 4: *E. coli* strains used in this study.

Supplementary Table 5: Comparison of characteristics of blue light-controlled *E. coli* expression systems.

Supplementary Figure 1.

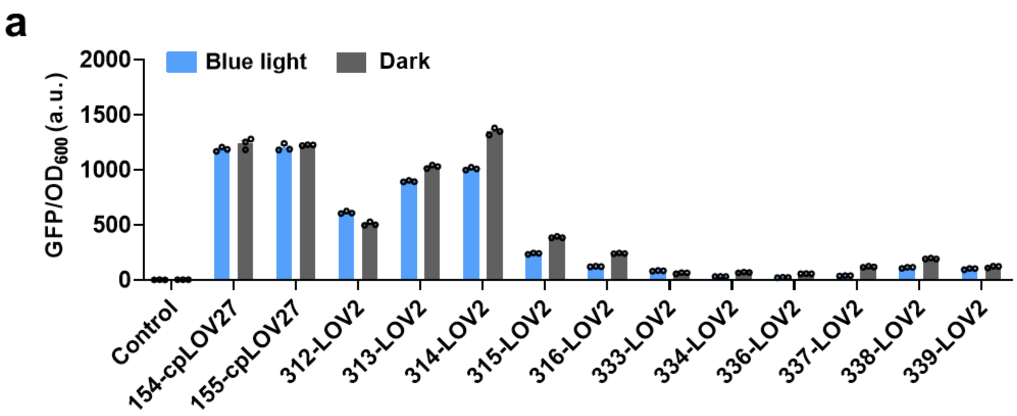

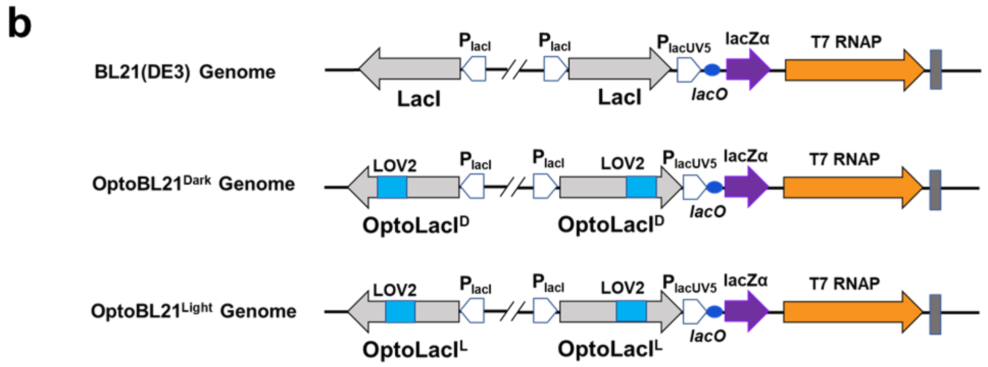

Supplementary Figure 2.

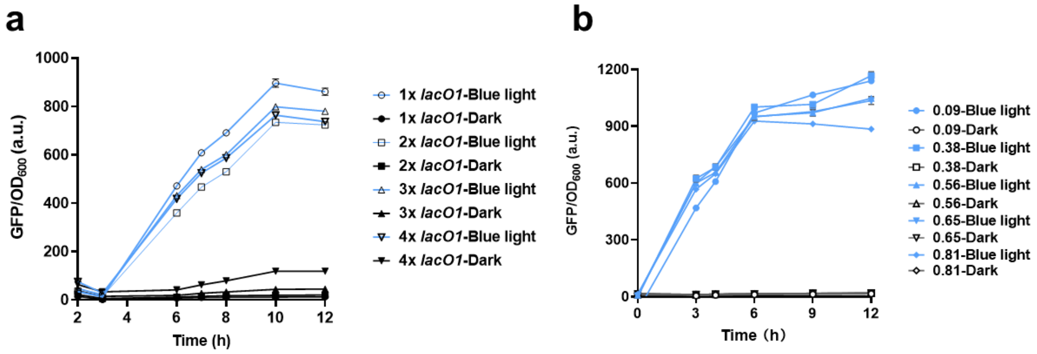

Supplementary Figure 3.

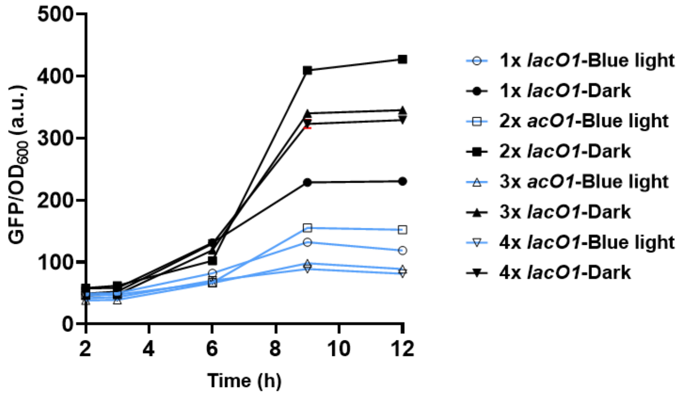

Supplementary Figure 4.

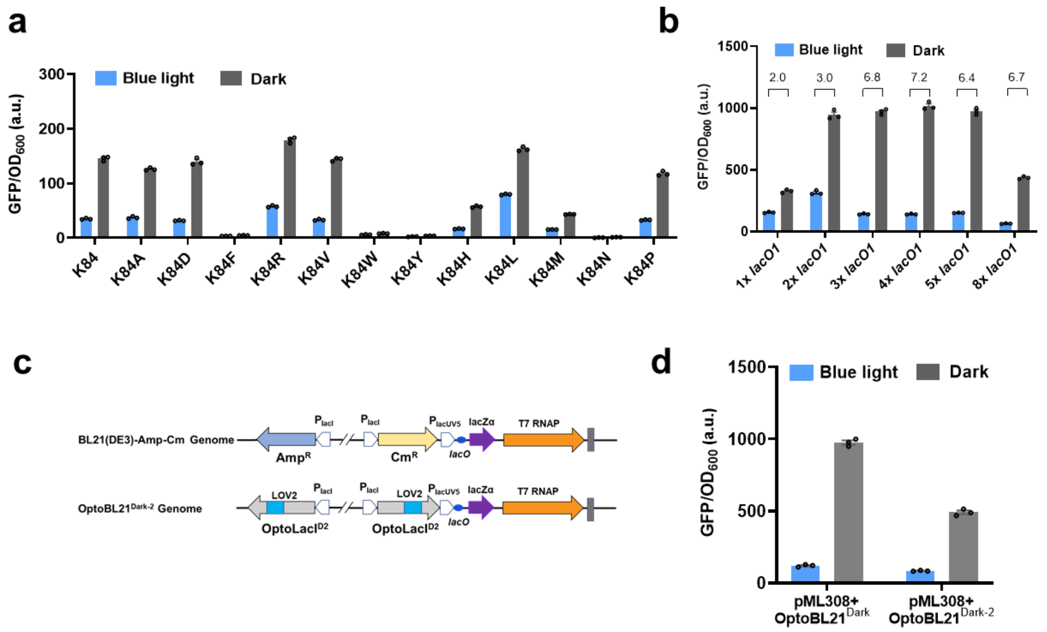

Supplementary Figure 5.

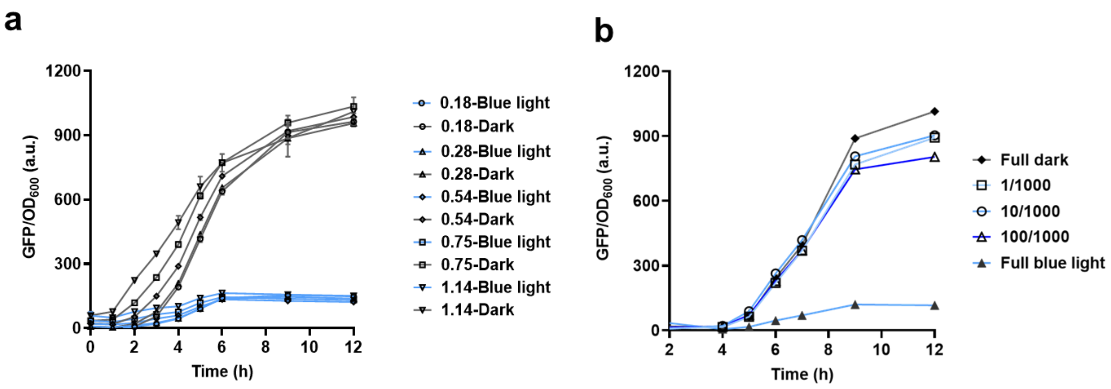

Supplementary Figure 6.

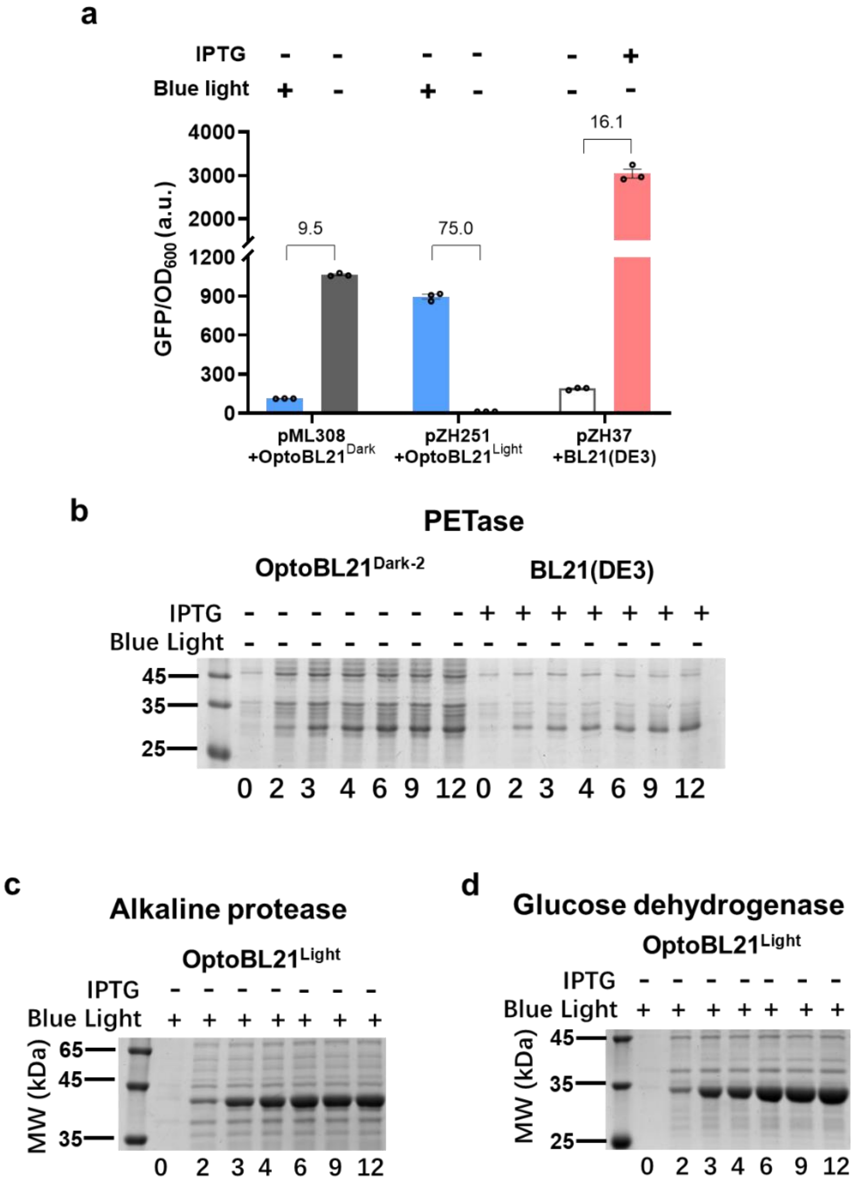

Supplementary Figure 7.

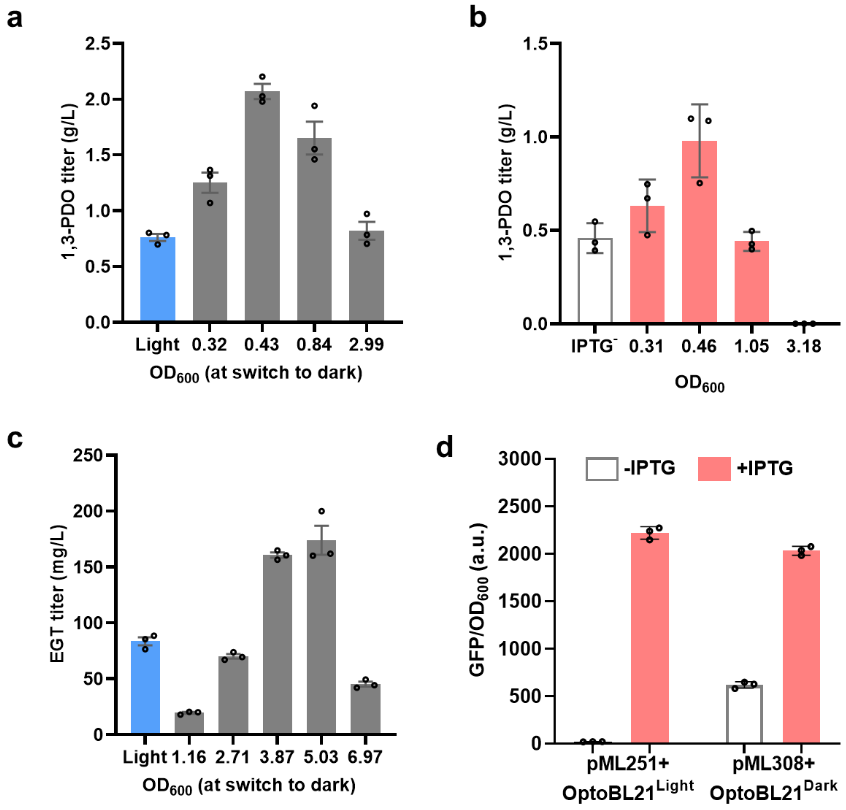

Supplementary Figure 8.

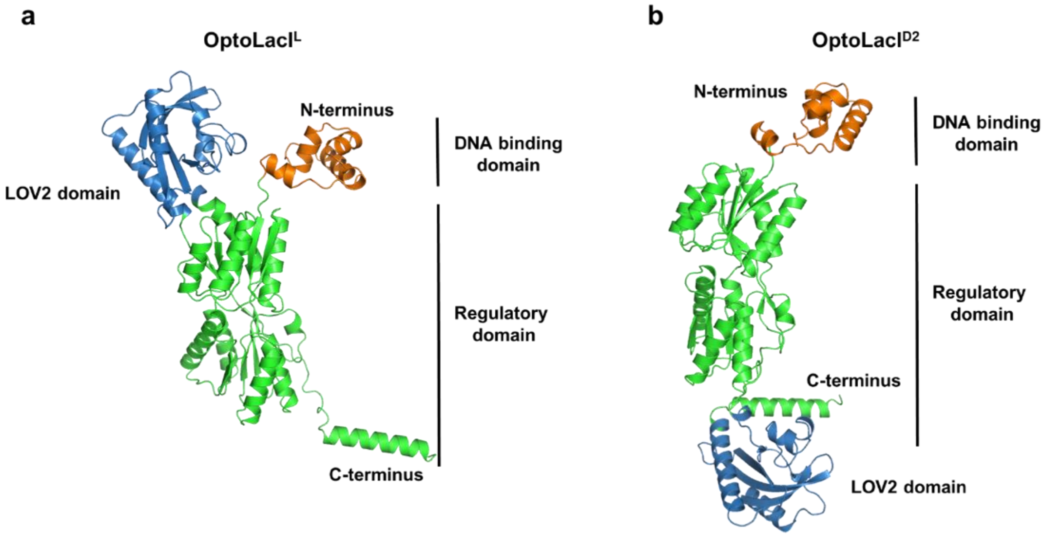

Supplementary Figure 9.

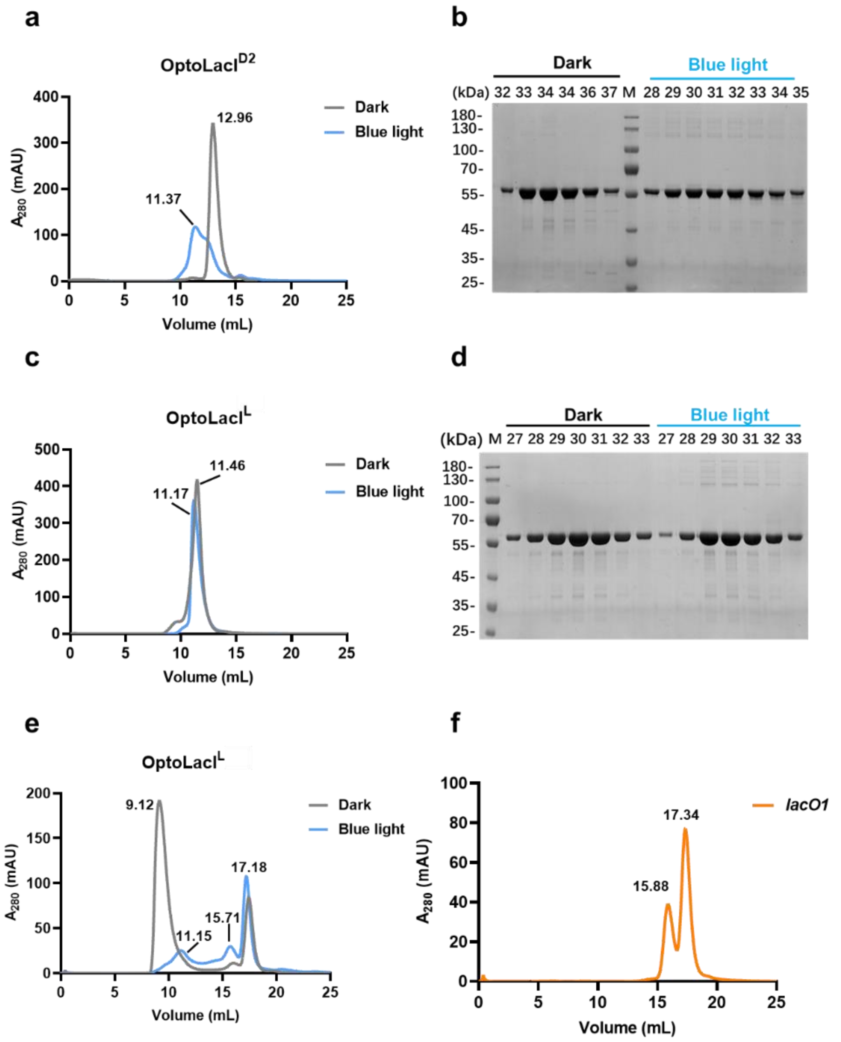

Supplementary Tables

Supplementary Table 1. Oligonucleotides used in this study.

Oligo Name	Sequence	Description
Zhl-Oli-190	GAAAACCGGACATGGCACTAAAGTCGCCTTCCC GTTCCGC	$LacI^{W220F}$-mutant-F
Zhl-Oli-191	GCGGAACGGGAAGGCGACTTTAGTGCCATGTCC GGTTTTC	$LacI^{W220F}$-mutant-R
Zhl-Oli-160	GAGAGGCGGTagctgcttcgtcgatgtttt	$LacI^{335}$-insert-LOV2-gene-Gibson-F
Zhl-Oli-161	CAATACGCAAttggaaagaattgaaaagaa	$LacI^{335}$-insert-LOV2- gene-Gibson-R
Zhl-Oli-162	ttctttccaaTTGCGTATTGGGCGCCAGGG	$LacI^{335}$-insert-LOV2-vector-Gibson-F
Zhl-Oli-163	cgaagcagctACCGCCTCTCCCCGCGCGTTG	$LacI^{335}$-insert-LOV2-vector-Gibson-R
Zhl-Oli-168	GGAATTGTGAGCGGATAACAATTCCTCTAGAAAT AATTTTGTTTAAC	2 x *lacO1*-mutant-F
Zhl-Oli-169	GGAATTGTTATCCGCTCACAATTCCGGGGAATTG TTATCCGCTCACaat	2 x *lacO1*-mutant-R
Zhl-Oli-170	AACAATTCCTCGGAATTGTGAGCGGATAACAATT CCTAGAAATAATTTTGTTTAAC	3 x *lacO1*-mutant-F
Zhl-Oli-171	CACAATTCCGAGGAATTGTTATCCGCTCACAATT CCGGGGAATTGTTATCCGCTCACAATTCCCCTATA	3 x *lacO1*-mutant-R
Zhl-Oli-407	TTAAGGAATTGTGAGCGGATAACAATTCCCCGGA ATTGTGAGCGGATAACAATTCCCCGGAATTGTGA	4 x *lacO1*-mutant-F
Zhl-Oli-408	aaagGGAATTGTTATCCGCTCACAATTCCGGGGAA TTGTTATCCGCTCACAATTCCGGGGAATTGTTATC	4 x *lacO1*-mutant-R
Zhl-Oli-230	CCTTCACCGCagctgcttcgtcgatgtttt	$LacI^{311}$-insert-LOV2-gene-Gibson-F
Zhl-Oli-231	TCAGGGCCAGttggaaagaattgaaaagaac	$LacI^{311}$-insert-LOV2-gene-Gibson-R
Zhl-Oli-232	ttctttccaaCTGGCCCTGAGAGAGTTGCAG	$LacI^{311}$-insert-LOV2-vector-Gibson-F
Zhl-Oli-233	cgaagcagctGCGGTGAAGGGCAATCAGCT	$LacI^{311}$-insert-LOV2-vector-Gibson-R
Zhl-Oli-250	CTCTGACCAGAGAGAAGGTGTTATGTTGAT	$LacI^{153}$-insert-cpLOV27-gene-Gibson-F

Zhl-Oli-251	TGATGGGTGTTTCTGCTGCATCTCTGACATG	$LacI^{153}$-insert-cpLOV27-gene-Gibson-R
Zhl-Oli-252	TGCAGCAGAAACACCCATCAACAGTATTAT	$LacI^{153}$-insert-cpLOV27-vector-Gibson-F
Zhl-Oli-253	CACCTTCTCTCTGGTCAGAGACATCAAGAAAT	$LacI^{153}$-insert-cpLOV27-vector-Gibson-R
Zhl-Oli-246	TGTCTCTGACAGAGAAGGTGTTATGTTGAT	$LacI^{152}$-insert-cpLOV27-gene-Gibson-F
Zhl-Oli-247	TGGGTGTCTGTTCTGCTGCATCTCTGACATG	$LacI^{152}$-insert-cpLOV27-gene-Gibson-R
Zhl-Oli-248	TGCAGCAGAACAGACACCCATCAACAGTAT	$LacI^{152}$-insert-cpLOV27-vector-Gibson-F
Zhl-Oli-249	CACCTTCTCTGTCAGAGACATCAAGAAATAAC	$LacI^{152}$-insert-cpLOV27-vector-Gibson-R
Zhl-Oli-491	TTGTCGCGGCGATTGAATCTCGCGCCGAT	$LacI^{K84E}$-mutant-F
Zhl-Oli-492	TCGGCGCGAGATTCAATCGCCGCGACAAT	$LacI^{K84E}$-mutant-R
Zhl-Oli-493	TTGTCGCGGCGATTTGCTCTCGCGCCGAT	$LacI^{K84C}$-mutant-F
Zhl-Oli-494	TCGGCGCGAGAGCAAATCGCCGCGACAAT	$LacI^{K84C}$-mutant-R
Zhl-Oli-473	TTGTCGCGGCGATTAGCTCTCGCGCCGAT	$LacI^{K84S}$-mutant-F
Zhl-Oli-474	TCGGCGCGAGAGCTAATCGCCGCGACAAT	$LacI^{K84S}$-mutant-R
Zhl-Oli-477	TTGTCGCGGCGATTACCTCTCGCGCCGATC	$LacI^{K84T}$-mutant-F
Zhl-Oli-478	ATCGGCGCGAGAGGTAATCGCCGCGACAAT	$LacI^{K84T}$-mutant-R
Zhl-Oli-467	ATTGTCGCGGCGATTATTTCTCGCGCCGATCAAC	$LacI^{K84I}$-mutant-F
Zhl-Oli-468	TGATCGGCGCGAGAAATAATCGCCGCGACAATTT	$LacI^{K84I}$-mutant-R
MZL-Oli-527	GCAGCGGCGCTAGCGCACAGCCTGCAAAAAAC	alkaline protease-gene-NheI-F
MZL-Oli-528	GTGGTGGTGCTCGAGTTACTGTGCTGCGGC	alkaline protease-gene-XhoI-R
MZL-Oli-545	CAGCAGCGGCGCTAGCATGTATCCTGATCTGAAAG	glucose dehydrogenase -gene-NheI-F

MZL-Oli-546	GGTGGTGGTGCTCGAGTTAACCACGACCCG	glucose dehydrogenase -gene-XhoI-R
MZL-Oli-794	GGCAGCAGCGTGAAACCAGTAACGTTATACGATGTCGC	LacI$^{335\text{-LOV2, W220F, K84E}}$/ LacI$^{311\text{-LOV2, W220F}}$-gene-Gibson-F
MZL-Oli-789	GTGGTGGTGCTGCCCGCTTTCCAGTCGG	LacI$^{335\text{-LOV2, W220F, K84E}}$/ LacI$^{311\text{-LOV2, W220F}}$-gene-Gibson-R
MZL-Oli-795	CTGGTTTCACGCTGCTGCCCATGGTATATCTCC	LacI$^{335\text{-LOV2, W220F, K84E}}$/ LacI$^{311\text{-LOV2, W220F}}$-vector-Gibson-R
MZL-Oli-788	GAAAGCGGGCAGCACCACCACCACCACCACTG	LacI$^{335\text{-LOV2, W220F, K84E}}$/ LacI$^{311\text{-LOV2, W220F}}$-vector-Gibson-F
JH-Oli-9	AGGCCTGTAGAAATAATTTTGTTTAACTTTAATAAGGAGATATACCATGAAAAGATCAAAACGATT	KpdhaB-KpgdrA-gene-Gibson-F
JH-Oli-10	CAGACTCGAGGATCTCCCACTGACCAAAGC	KpdhaB-KpgdrA-gene-Gibson-R
JH-Oli-1	AGGCCTGTAGAAATAATTTTGTTTAACTTTAATAAGGAGATATACCATGCAACACAACCTATTGATA	EcyqhD-gene-Gibson-F
JH-Oli-2	AGCGACATGACGGTCCCCTCTTAGCTATGCTTAACCATCAC	EcyqhD-gene-Gibson-R
JH-Oli-3	GAGGGGACCGTCATGTCGCT	KpgdrB-gene-Gibson-F
JH-Oli-4	GCTCGAATTCTCAGTTTCTCTCACTTAACG	KpgdrB-gene-Gibson-R
JY-Oli-645	CTTTAAGAAGGAGATATAATGAGCATGAGCTTTAGCCTG	Egt1-gene-Gibson-F
JY-Oli-648	TATATCTCCTTCTTAAAGTTAAACAAACCATGGCAGATCGCGCACCACGCGCG	Egt1-gene-Gibson-R
JY-Oli-647	TGGTTTGTTTAACTTTAAGAAGGAGATATAATGACCCTGAGCTTAGCGAAC	EgtD-gene-Gibson-F
JY-Oli-650	GATGATGGCTGCTGCGCGCACCGCCAGGCTCAGGC	EgtD/EgtE-gene-Gibson-R
JY-Oli-651	TTAAGAAGGAGATATAATGATGCTGGCGCAGCAGTG	EgtE-gene-Gibson-F

Supplementary Table 2. Amino acid sequences used in this study.

**Protein name**	**Sequence**
AsLOV2	LERIEKNFVITDPRLPDNPIIFASDSFLQLTEYSREEILGRNCRFLQGPETDRATVR<br>KIRDAIDNQTEVTVQLINYTKSGKKFWNLFHLQPMRDQKGDVQYFIGVQLDG<br>TEHVRDAAEREGVMLIKKTAENIDEAA
cpLOV27	REGVMLIKKTAENIDEAAKELGGGSGGSLATTLERIEKNFVITDPRLPDNPIIFAS<br>DSFLQLTEYSREEILGRNCRFLQGPETDRATVRKIRDAIDNQTEVTVQLINYTKS<br>GKKFWNLFHLQPMRDQKGDVQYFIGVQLDGTEHVRDAAE
sfGFP	MSKGEELFTGVVPILVELDGDVNGHKFSVRGEGEGDATIGKLTLKFICTTGKLP<br>VPWPTLVTTLTYGVQCFSRYPDHMKRHDFFKSAMPEGYVQERTISFKDDGKY<br>KTRAVVKFEGDTLVNRIELKGTDFKEDGNILGHKLEYNFNSHNVYITADKQKN<br>GIKANFTVRHNVEDGSVQLADHYQQNTPIGDGPVLLPDNHYLSTQTVLSKDPN<br>EKRDHMVLHEYVNAAGIT
PETase	QTNPYARGPNPTAASLEASAGPFTVRSFTVSRPSGYGAGTVYYPTNAGGTVGA<br>IAIVPGYTARQSSIKWWGPRLASHGFVVITIDTNSTLDQPSSRSSQQMAALRQV<br>ASLNGTSSSPIYGKVDTARMGVMGWSMGGGGSLISAANNPSLKAAAPQAPW<br>DSSTNFSSVTVPTLIFACENDSIAPVNSSALPIYDSMSRNAKQFLEINGGSHSCA<br>NSGNSNQALIGKKGVAWMKRFMDNDTRYSTFACENPNSTRVSDFRTANCS
alkaline protease	AQPAKNVEKDYIVGFKSGVKTASVKKDIIKESGGKVDKQFRIINAAKAKLDKE<br>ALKEAKNDPDVAYVEEDHVAHALAQTVPYGIPLIKADKLHAQGFKGANVKGA<br>VLATGIPTSHPDLNVVGGASFVAGEAYNTDGNGHGTHVAGTVAALDNTTGVL<br>GVAPSVSLYAVKVLNSSGSGSYSGIVSGIEWATTNGMDVINMSLGGASGSTAMK<br>QAVDNAYAKGVVVVAAAGNSGSSGNTNTIGYPAKYDSVIAVGAVDSNSNRASF<br>SSVGAELEVMAPGAGVYSTYPTNTYATLNGTSMASPHVAGAAALILSKHPNLS<br>ASQVRNRLSSTATYLGSSFYYGKGLINVEAAAQ*
glucose dehydrogenase	MYPDLKGKVVAITGAASGLGKAMAIRFGKEQAKVVINYYSNKQDPNEVKEEV<br>IKAGGEAVVVQGDVTKEEDVKNIVQTAIKEFGTLDIMINNAGLENPVPSHEMPL<br>KDWDKVIGTNLTGAFLGSREAIKYFVENDIKGNVINMSSVHEVIPWPLFVHYA<br>ASKGGIKLMTETLALEYAPKGIRVNNIGPGAINTPINAEKFADPKQKADVESMIP<br>MGYIGEPEEIAAVAAWLASKEASYVTGITLFADGGMTQYPSFQAGRG*

KpgdrA	MPLIAGIDIGNATTEVALASDDPQARAFVASGIVATTGMKGTRDNIAGTLAALE<br>QALAKTPWSMSDVSRIYLNEAAPVIGDVAMETITETIITESTMIGHNPQTPGGV<br>GVGVGTTIALGRLATLPAAQYAEGWIVLIDDAVDFLDAVWWLNEALDRGINV<br>VAAILKKDDGVLVNNRLRKTLPVVDEVTLLEQVPEGVMAAVEVAAPGQVVRI<br>LSNPYGIATFFGLSPEETQAIVPIARALIGNRSAVVLKTPQGDVQSRVIPAGNLYI<br>SGEKRRGEADVAEGAEAIMQAMSACAPVRDIRGEPGTHAGGMLERVRKVMA<br>SLTDHEMSAIYIQDLLAVDTFIPRKVQGGMAGECAMENAVGMAAMVKADRL<br>QMQVIARELSARLQTEVVVGGVEANMAIAGALTTPGCAAPLAILDLGAGSTD<br>AAIVNAEGQITAVHLAGAGNMVSLLIKTELGLEDLSLAEAIKKYPLAKVESLFSI<br>RHENGAVEFFREALSPAVFAKVVYIKEGELVPIDNASPLEKIRLVRRQAKEKVFV<br>TNCLRALRQVSPGGSIRDIAFVVLVGGSSLDFEIPQLITEALSHYGVVAGQGNIR<br>GTEGPRNAVATGLLLAGQAN*
KpdhaB1	MKRSKRFAVLAQRPVNQDGLIGEWPEEGLIAMDSPFDPVSSVKVDNGLIVELD<br>GKRRDQFDMIDRFIADYAINVERTEQAMRLEAVEIARMLVDIHVSREEIIAITTAI<br>TPAKAVEVMAQMNVVEMMMALQKMRARRTPSNQCHVTNLKDNPVQIAADA<br>AEAGIRGFSEQETTVGIARYAPFNALALLVGSQCGRPGVLTQCSVEEATELELG<br>MRGLTSYAETVSVYGTEAVFTDGDDTPWSKAFLASAYASRGLKMRYTSGTGSE<br>ALMGYSESKSMLYLESRCIFITKGAGVQGLQNGAVSCIGMTGAVPSGIRAVLAE<br>NLIASMLDLEVASANDQTFSHSDIRRTARTLMQMLPGTDFIFSGYSAVPNYDNM<br>FAGSNFDAEDFDDYNILQRDLMVDGGLRPVTEAETIAIRQKAARAIQAVFRELG<br>LPPIADEEVEAATYAHGSNEMPPRNVVEDLSAVEEMMKRNITGLDIVGALSRS<br>GFEDIASNILNMLRQRVTGDYLQTSAILDRQFEVVSAVNDINDYQGPGTGYRIS<br>AERWAEIKNIPGVVQPDTIE*
KpdhaB2	VQQTTQIQPSFTLKTREGGVASADERADEVVIGVGPAFDKHQHHTLIDMPHGAI<br>LKELIAGVEEEGLHARVVRILRTSDVSFMAWDAANLSGSGIGIGIQSKGTTVIH<br>QRDLLPLSNLELFSQAPLLTLETYRQIGKNAARYARKESPSPVPVVNDQMVRPK<br>FMAKAALFHIKETKHVVQDAEPVTLHVDLVRE*
KpdhaB3	MSEKTMRVQDYPLATRCPEHILTPTGKPLTDITLEKVLSGEVGPQDVRISCQTLE<br>YQAQIAEQMQRHAVARNFRRAAELIAIPDERILAIYNALRPFRSSQAELLAIADE<br>LEHTWHATVNAAFVRESAEVYQQRHKLRKGS*
KpgdrB	MSLSPPGVRLFYDPRGHHAGAINELCWGLEEQGVPCQTITYDGGGDAAALGA<br>LAARSSPLRVGIGLSASGEIALTHAQLPADAPLATGHVTDSDDHLRTLGANAGQ<br>LVKVLPLSERN*

EcyqhD	MNNFNLHTPTRILFGKGAIAGLREQIPHDARVLITYGGGSVKKTGVLDQVLDA<br>LKGMDVLEFGGIEPNPAYETLMNAVKLVREQKVTFLLAVGGGSVLDGTKFIAA<br>AANYPENIDPWHILQTGGKEIKSAIPMGCVLTLPATGSESNAGAVISRKTTGDK<br>QAFHSAHVQPVFAVLDPVYTYTLPPRQVANGVVDAFVHTVEQYVTKPVDAKI<br>QDRFAEGILLTLIEDGPKALKEPENYDVRANVMWAATQALNGLIGAGVPQDW<br>ATHMLGHELTAMHGLDHAQTLAIVLPALWNEKRDTKRAKLLQYAERVWNITE<br>GSDDERIDAAIAATRNFFEQLGVPTHLSDYGLDGSSIPALLKKLEEHGMTQLGE<br>NHDITLDVSRRIYEAAR*
Egt1	MSMSFSLIPSVYARSALPTLDDWEALWATWDVVTRQMLPQEELLEKPIKLRNA<br>CIFYLGHIPTFLDIQLTKTTKQAPSEPAHFCKIFERGIDPDVDNPELCHAHSEIPAE<br>WPPVEEILTYQETVRSRLRGLYAHGIANIPRNVGRAIWVGFEHELMHIETLLYM<br>MLQSDKTLIPTHIPRPDFDKLARKAESERVPNQWFKIPAQEITIGLDDPEDGSDI<br>NKHYGWDNEKPPRRVQVAAFQAQGRPITNEEYAQYLLEKNIDKLPASWARLD<br>NENISNGTTNSVSGHHSNRTSKQQLPSSFLEKTAVRTVYGLVPLKHALDWPVFA<br>SYDELAGCAAYMGGRIPTFEETRSIYAYADALKKKKEAERQLGRTVPAVNAHL<br>TNNGVEITPPSSPSSETPAESSSPSDSNTTLITTEDLFSDLDGANVGFHNWHPMPI<br>TSKGNTLVGQGELGGVWEWTSSVLRKWEGFEPMELYPGYTADFFDEKHNIVL
EgtD	MTLSLANYLAADSAAEALRRDVRAGLTAAPKSLPPKWFYDAVGSDLFDQATR<br>LPEYYPTRTEAQILRTRSAEIIAAAGADTLVELGSGTSEKTRMLLDAMRDAELL<br>RRFIPFDVDAGVLRSAGAAIGAEYPGIEIDAVCGDFEEHLGKIPHVGRRLVVFLG<br>STIGNLTPAPRAEFLSTLADTLQPGDSLLLGTDLVKDTGRLVRAYDDAAGVTAA<br>FNRNVLAVVNRELSADFDLDAFEHVAKWNSDEERIEMWLRARTAQHVRVAAL<br>DLEVDFAAGEEMLTEVSCKFRPENVVAELAEAGLRQTHWWTDPAGDFGLSLA<br>VR
EgtE	MMLAQQWRDARPKVAGLHLDSGACSRQSFAVIDATTAHARHEAEVGGYVAA<br>EAATPALDAGRAAVASLIGFAASDVVYTSGSNHAIDLLLSSWPGKRTLACLPGE<br>YGPNLSAMAANGFQVRALPVDDDGRVLVDEASHELSAHPVALVHLTALASHR<br>GIAQPAAELVEACHNAGIPVVIDAAQALGHLDCNVGADAVYSSSRKWLAGPR<br>GVGVLAVRPELAERLQPRIPPSDWPIPMSVLEKLELGEHNAAARVGFSVAVGEH<br>LAAGPTAVRERLAEVGRLSRQVLAEVDGWRVVEPVDQPTAITTLESTDGADPA<br>SVRSWLIAERGIVTTACELARAPFEMRTPVLRISPHVDVTVDELEQFAAALREA<br>P

Supplementary Table 3. Plasmids used in this study.

Plasmid	Description	Source
pET-28a	Kan^R, P_{T7}- 1x *lacO1*-MCS-T_{T7} -P_{LacI}-LacI	Novagen
pZH35	Kan^R, P_{T7}- 3x *lacO1*-sfGFP-T_{T7} -P_{LacI}-LacI ^{W220F}	This study
pZH36	Kan^R, P_{T7}- 3x *lacO1*-sfGFP-T_{T7}-P_{LacI}-$LacI^{335\text{-}LOV2,\ W220F}$	This study
pZH37	Kan^R, P_{T7}- 1x *lacO1*-sfGFP-T_{T7} -P_{LacI}-LacI	This study
pZH58	Kan^R, P_{T7}- 3x *lacO1*-sfGFP-T_{T7} -P_{LacI}-$LacI^{311\text{-}LOV2,\ W220F}$	This study
pZH76	Sm^R, Upstream 500 $bp^{LacI\text{-}1}$-$LacI^{335\text{-}LOV2,\ W220F}$- Downstream 500 bp $^{LacI\text{-}1}$-P_{J23119}-gRNA scaffold	This study
pZH78	Sm^R, Upstream 500 $bp^{LacI\text{-}2}$-$LacI^{335\text{-}LOV2,\ W220F}$- Downstream 500 bp $^{LacI\text{-}2}$-P_{J23119}-gRNA scaffold	This study
pZH83	Sm^R, P_{J23119}-N20-$1^{LacI\text{-}335\text{-}LOV2,\ W220F}$-gRNA scaffold- P_{J23119}-N20-2 $^{LacI\text{-}335\text{-}LOV2,\ W220F}$ -gRNA scaffold	This study
pZH91	Kan^R, P_{T7}- 1x *lacO1*-sfGFP-T_{T7} -P_{LacI}-$LacI^{335\text{-}LOV2,\ W220F}$	This study
pZH96	Kan^R, P_{T7}- 2x *lacO1*-sfGFP-T_{T7} -P_{LacI}-$LacI^{335\text{-}LOV2,\ W220F}$	This study
pZH99	Kan^R, P_{T7}- 4x *lacO1*-sfGFP-T_{T7} -P_{LacI}-$LacI^{335\text{-}LOV2,\ W220F}$	This study
pZH151	Kan^R, P_{T7}- 3x *lacO1*-sfGFP-T_{T7} -P_{LacI}-$LacI^{335\text{-}LOV2,\ W220F,\ K84E}$	This study
pZH251	Kan^R, P_{T7}- 1x *lacO1*-sfGFP-T_{T7}-P_{LacI}-$LacI^{311\text{-}LOV2,\ W220F}$	This study
pZH261	Kan^R, P_{T7}- 2x *lacO1*-sfGFP-T_{T7} -P_{LacI}-$LacI^{311\text{-}LOV2,\ W220F}$	This study

pZH262	Kan^R, P_{T7}- 4x *lacO1*-sfGFP-T_{T7} -P_{LacI}-$LacI^{311\text{-}LOV2,\ W220F}$	This study
pML290	Kan^R, P_{T7}- 2x *lacO1*-sfGFP-T_{T7} -P_{LacI}-$LacI^{335\text{-}LOV2,\ W220F,\ K84E}$	This study
pML291	Kan^R, P_{T7}- 1x *lacO1*-sfGFP-T_{T7}-P_{LacI}-$LacI^{335\text{-}LOV2,\ W220F,\ K84E}$	This study
pML308	Kan^R, P_{T7}- 4x *lacO1*-sfGFP-T_{T7} -P_{LacI}-$LacI^{335\text{-}LOV2,\ W220F,\ K84E}$	This study
pML310	Kan^R, P_{T7}- 5x *lacO1*-sfGFP-T_{T7} -P_{LacI}-$LacI^{335\text{-}LOV2,\ W220F,\ K84E}$	This study
pML313	Kan^R, P_{T7}- 8x *lacO1*-sfGFP-T_{T7} -P_{LacI}-$LacI^{335\text{-}LOV2,\ W220F,\ K84E}$	This study
pML333	Kan^R, P_{T7}- 4x *lacO1*-alkaline protease-T_{T7} -P_{LacI}-$LacI^{335\text{-}LOV2,\ W220F,\ K84E}$	This study
pML351	Kan^R, P_{T7}- 4x *lacO1*-PETase-T_{T7} -P_{LacI}-$LacI^{335\text{-}LOV2,\ W220F,\ K84E}$	This study
pML353	Kan^R, P_{T7}- 4x *lacO1*-glucose dehydrogenase-T_{T7} -P_{LacI}-$LacI^{335\text{-}LOV2,\ W220F,\ K84E}$	This study
pML318	Sm^R, Upstream 500 $bp^{LacI\text{-}1}$-$LacI^{335\text{-}LOV2,\ W220F,\ K84E}$-Downstream 500 $bp^{LacI\text{-}1}$-P_{J23119}-gRNA scaffold	This study
pML320	Sm^R, Upstream 500 $bp^{LacI\text{-}2}$-$LacI^{335\text{-}LOV2,\ W220F,\ K84E}$-Downstream 500 $bp^{LacI\text{-}2}$-P_{J23119}-gRNA scaffold	This study
pML340	Sm^R, P_{J23119}-N20-$1^{AmpR,}$ -gRNA scaffold- P_{J23119}-N20-2^{CamR} -gRNA scaffold	This study
pML347	Sm^R, Upstream 500 $bp^{LacI\text{-}1}$-$LacI^{311\text{-}LOV2,\ W220F}$- Downstream 500 $bp^{LacI\text{-}1}$-P_{J23119}-gRNA scaffold	This study
pML348	Sm^R, Upstream 500 $bp^{LacI\text{-}2}$-$LacI^{311\text{-}LOV2,\ W220F}$- Downstream 500 $bp^{LacI\text{-}2}$-P_{J23119}-gRNA scaffold	This study
pML363	Kan^R, P_{T7}- 4x *lacO1*-$LacI^{335\text{-}LOV2,\ W220F,\ K84E}$- T_{T7} -P_{LacI}-$LacI^{335\text{-}LOV2,\ W220F,\ K84E}$	This study
pML364	Kan^R, P_{T7}- 3x *lacO1*-$LacI^{311\text{-}LOV2,\ W220F}$- T_{T7} -P_{LacI}-$LacI^{311\text{-}LOV2,\ W220F}$	This study

pML409	Kan^R, P_{T7}-1x *lacO1*-alkaline protease-T_{T7}-P_{LacI}-$LacI^{311-LOV2, W220F}$	This study
pML410	Kan^R, P_{T7}-1x *lacO1*-glucose dehydrogenase-T_{T7}-P_{LacI}-$LacI^{311-LOV2, W220F}$	This study
pJH10	Sm^R, P_{T7} -1x *lacO1*- EcyqhD- KpgdrB - T_{T7}-P_{T7}-KpdhaB KpgdrA-T_{T7}-P_{LacI}-LacI	This study
pJH11	Sm^R, P_{T7} -1x *lacO1*- EcyqhD- KpgdrB - T_{T7}-P_{T7}-KpdhaB KpgdrA-T_{T7}-P_{LacI}-$LacI^{335-LOV2, W220F}$	This study
pJY111	Kan^R, P_{T7} - 1x *lacO1*- Egt1- EgtD - T_{T7}-P_{T7}- EgtE - T_{T7}-P_{LacI}-LacI	This study
pJY165	Kan^R, P_{T7}-1x *lacO1*- Egt1- EgtD - T_{T7}-P_{T7}- EgtE - T_{T7}-P_{LacI}-$LacI^{335-LOV2, W220F, K84E}$	This study

Supplementary Table 4. *E. coli* strains used in this study.

Strain	Description	Genotype	Source
Escherichia coli DH5α	*E.coli* strain for routine transformations.	*F⁻endA1 glnV44 thi-1 recA1 relA1 gyrA96 deoR nupG purB20 φ80dlacZΔM15 Δ(lacZYA-argF)U169, hsdR17(rK⁻mK⁺), λ⁻*	Lab stock
Escherichia coli BL21(DE3)	A widely used T7 expression *E. coli* strain	*E.coli* B F⁻ *dcm ompT lon hsd*S(r_B^-, m_B^-,) *gal* λ(DE3 [*lacI lacUV5-T7p07 ind1 sam7 nin5*]) [*malB*⁺]$_{K-12}$($λ^S$)	Lab stock
OptoBL21^{Light}	OptoBL21 strain used for blue light-induced gene expression. (Two copies of *lacI* were both substituted with *optolacI*^{L})	BL21(DE3)-Δ(*lacI*)::*lacI*$^{311\text{-}LOV2, W220F}$, 335309,749140	This study
OptoBL21^{Dark}	OptoBL21 strain used for dark-induced gene expression (Two copies of *lacI* were both substituted with *optolacI*^{D})	BL21(DE3)-Δ(*lacI*)::*lacI*$^{335\text{-}LOV2, W220F}$, 335309,749140	This study
OptoBL21$^{Dark\text{-}2}$	OptoBL21 strain used for dark-induced gene expression (Two copies of *lacI* were both substituted with *optolacI*^{D2})	BL21(DE3)-Δ(*lacI*)::*lacI*$^{335\text{-}LOV2,W220F, K84E}$, 335309,749140	This study

Supplementary Table 5. Comparison of characteristics of blue light-controlled *E. coli* expression systems

**Blue light-controlled *E. coli* expression systems**	**OptoLAC system**	**BLADE system**	**OptoLacI and Opto*E.coli* systems**
**Target for optogenetic engineering**	*lac* operon (the expression of LacI)	L-arabinose-responsive protein AraC	Lactose repressor protein LacI
**Photosensory modules**	pDawn system (photosensory histidine kinase YF1)	Vivid (VVD) domain	LOV2 domain
**Principles of optogenetic control**	Control of the *lac* operon (by controlling the expression of LacI) using the blue light-triggered pDawn system	AraC dimerization triggered by blue light	LacI conformational changes induced by blue light or darkness
**Engineering strategy**	Optogenetic engineering of a gene circuit (opto-gene circuit engineering)	Optogenetic protein engineering (opto-protein engineering)	Optogenetic protein engineering (opto-protein engineering)
**Core components**	Four proteins (YF1, FixJ, cI, and LacI) and three layers of logic gates	AraC-VVD fusion chimera	LacI-LOV2 insertion chimera
**Induction methods**	Dark	Blue light	Dark or blue light
**Reference**	*Nat Chem Biol* 17, 71–79 (2021)	*Nat Chem Biol* 17, 817–827 (2021)	This work
